# Beyond smell and taste: the role of mouthfeel in flavour perception and its implications for health and food innovation

**DOI:** 10.1038/s41538-026-01232-5

**Published:** 2026-11-10

**Authors:** Julia Benthin, Marina Wiesenfarth, Rita dos Santos Natividade, David Duria, Sanjai Karanth, Melanie Koehler

**Affiliations:** 1 Leibniz Institute for Food Systems Biology at the Technical University of Munich, Lise-Meitner-Str. 34, Freising, Germany; 2 TUM Graduate School, TUM School of Life Sciences Weihenstephan, Technical University of Munich, Alte Akademie 8, Freising, Germany; 3 TUM Junior Fellow, TUM School of Life Sciences Weihenstephan, Technical University of Munich, Freising, Germany

**Keywords:** Engineering, Neuroscience

## Abstract

Mouthfeel, encompassing the tactile, thermal, and chemesthetic sensations experienced during eating and drinking, is a crucial dimension of flavour perception. This review includes insights from sensory science, neurophysiology, biophysics, molecular biology, and food technology to provide a mechanistic perspective on mouthfeel and its relevance to nutrition and product development. We discuss how integrating this perspective with food design may enable better strategies to promote healthier and more satisfying diets.

## Main

**Fig. 1:**
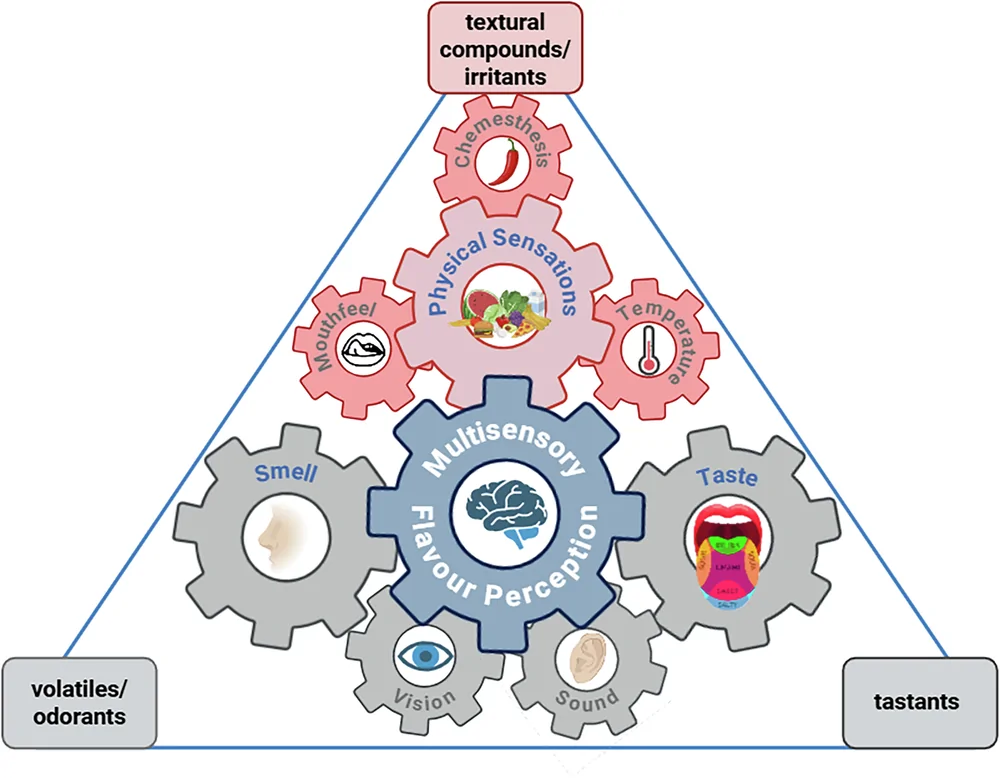
Food flavour triangle–integration of various physical sensations as key pillars. Smell, taste and physical sensations are shown as the most important and equally contributing key pillars in the multisensory perception of flavour, whereas vision and sound are less dominant^76^. Mouthfeel, chemesthesis, and temperature are categorized as physical sensations because they share similar trigeminal pathways, yet they differ perceptibly. Created in BioRender. https://BioRender.com/71z349n.

The flavour of food is important in our daily lives, both nutritionally and culturally. During eating, consumers judge a complex combination of components that are sensed in the nose as aroma (primarily from volatile compounds), on the tongue as taste, and in the oral cavity as texture and mouthfeel^1^. These sensory modalities arise from both volatile and non-volatile compounds released during consumption and can be broadly categorised as tastants, odorants, and textural compounds/irritants^2,3^. Food texture is considered a multidimensional sensory property given by the food’s structure, rheology, and surface properties^4^. Mouthfeel, on the other hand, is a term often used to refer to the tactile aspects of texture perception during consumption^5–7^. Figure 1 illustrates the integration of physical sensations, such as mouthfeel, temperature, and chemesthesis within the multisensory framework of food flavour perception.

Texture itself is defined as 'all the rheological and structural (geometrical and surface) attributes of a food product perceptible by mechanical, tactile, and where appropriate, visual, and auditory receptors' (ISO 5492:2008). It encompasses how food feels in the mouth, such as crispiness, smoothness, creaminess, or chewiness, as well as how its structure changes during mastication^8^. Saliva plays an important role during oral processing. Secreted in response to taste, aroma, and mechanical stimulation, saliva contributes to food breakdown and modulates perceived texture^9^. For example, salivary amylase reduces starch viscosity during consumption, while dilution of thickening agents can alter both texture and taste perception^10^. As food transforms into a bolus upon entering the mouth, changes in structure and lubrication continuously reshape sensations such as thickness and smoothness^5^. Because these evolving oral cues influence product evaluation and intake decisions^11–15^, understanding oral processing is essential for designing foods that are both appealing and supportive of healthier food choices.

A primary reason for rejecting foods or beverages is dissatisfaction with their flavour. Although often overlooked, mouthfeel is a central component of flavour perception and a key determinant of liking, acceptance, and overall consumption experience^11,15^. The perception of mouthfeel is driven by the chemical and physical activation of cell receptors in the oral cavity, generating sensory signals that are transmitted via the trigeminal nervous system to the brain^16^. The pleasure derived through food textures can be linked to tactile enjoyment from skin contact: individual differences in tactile sensitivity, shaped by detection thresholds, prior experiences, and culture, translate into distinct texture preferences^17^. Across many contexts, crispy and crunchy textures are generally preferred over tough, gummy, or slimy ones^4,15^. In beverages and dairy systems, 'body' is described as perceived weight or heaviness, and similarly contributes to quality judgements and consumer choice, as exemplified in coffee and wine evaluation^18^. Such variations in mouthfeel perception not only drive purchasing intent but are also associated with selective or picky eating behaviours^16,19^.

By shaping food preference and consumer choice, mouthfeel also influences/ modulates dietary and eating behaviour, and metabolic responses to food.^14,15,20^. Food texture affects eating rate, influencing energy intake to satiation, thereby exerting a direct impact on health^12,20–23^. A higher eating rate, for example, has been linked to obesity and adverse cardiometabolic health^24^. As public health guidelines recommend reductions in sugar, salt, and fat, it is essential to account for the sensory consequences in product formulation. Therefore, a mechanistic understanding of sensory cues can support the development of reformulated foods and beverages that promote healthier eating behaviours without compromising acceptance^25,26^.

This review will focus on the role of texture and mouthfeel in health and discuss how understanding their perception and physiological mechanisms can inform food design and public health strategies. In order to elucidate the vital yet underexplored contribution of mouthfeel in flavour perception, we first describe its physiological basis known to date, outline the underlying somatosensory pathways and their integration in the brain, as well as sensory descriptors alongside psychological factors.

## Physiology of mouthfeel

**Fig. 2:**
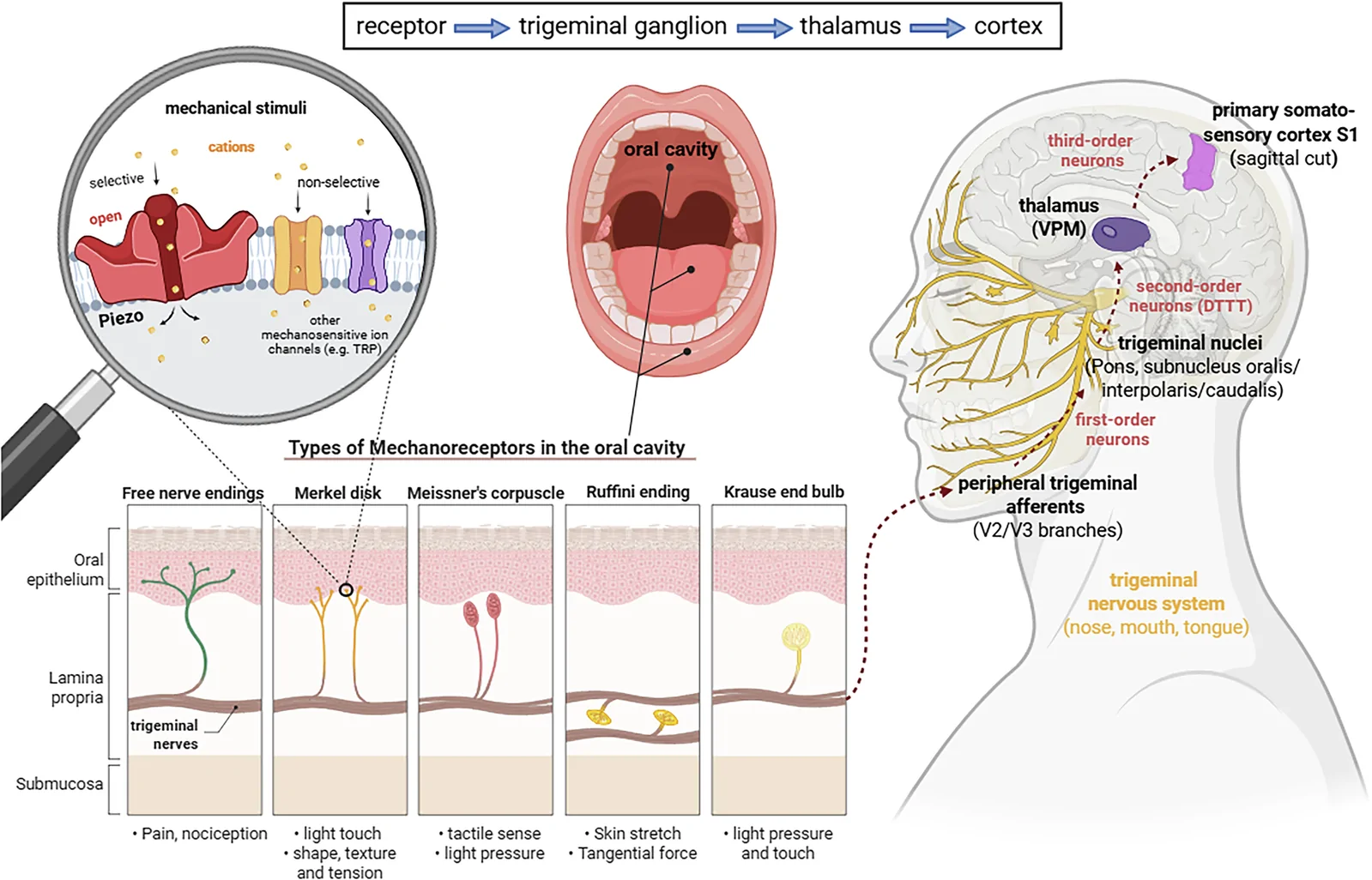
Physiology of mouthfeel, including the anatomy and location of mechanoreceptors in the oral cavity, as well as the trigeminal pathway to the brain. Mechanoreceptors are present all over the oral cavity (tongue, oral mucosa, and lips). The schematic overview of how mechanical stimuli are transmitted via the trigeminal nervous system, including the specialised dorsal trigeminothalamic tract (DTTT), to the ventral posteromedial nucleus (VPM) of the thalamus and the primary sensory cortex (S1) is shown on the right. Created in BioRender. https://BioRender.com/71z349n.

Mouthfeel can be viewed as a neural symphony: multiple peripheral inputs in the oral cavity converge and are integrated with brain circuits for memory, emotion, and reward, shaping individual eating behaviour and food choice^27,28^. This diversity of signals is enabled by the structure of the oral cavity itself, which comprises multiple epithelial and subepithelial tissues (tongue, oral mucosa, hard palate, and gingiva) that differ in compliance, surface composition, and innervation density^29^. Functionally, mouthfeel emerges from both chemesthetic sensations (e.g., burning, cooling, and tingling) and somatosensory sensations (e.g. pressure, touch, temperature, and pain)^30^. Crucially, mechanoreceptors do not detect 'texture' per se; they detect physical forces generated during oral intake and processing, such as membrane deformation and tension, indentation, shear, vibration, and slip, which they convert into electrical activity^9^. In the trigeminal system, low-threshold mechanosensation is commonly described in terms of response dynamics rather than anatomy: rapidly adapting (RA) receptors preferentially encode stimulus onset and change (movement, vibration, slip), whereas slowly adapting (SA) receptors encode sustained contact and deformation (pressure, stretch). Within the oral cavity, these functional classes include SA I (often Merkel cell-associated), SA II (Ruffini-like/stretch-sensitive), and RA (Meissner-like) receptors, alongside deeper afferents that likely contribute proprioceptive information^31^. At the molecular level, force-to-signal conversion is mediated by mechanosensitive ion channels embedded in the membrane of these sensory endings and associated epithelial cells. Mechanical stress promotes channel opening, cation influx (notably Na⁺ and Ca²⁺), depolarisation and receptor potentials, which—when sufficient—trigger action potentials that propagate along trigeminal afferents to central circuits where oral texture is ultimately perceived and integrated. Candidates for these primary transducers include Piezo channels (key mechanotransducers in touch-related signalling) as well as subsets of transient receptor potential (TRP) channels (e.g., some TRPV, TRPA, TRPM, and TRPP members), while free nerve endings and additional ion channels (including proton-sensitive and selective potassium/sodium channels) contribute to broader oral somatosensation, including nociception and chemesthesis^32^. The anatomy and location of mechanoreceptors in the oral cavity are shown in Fig. 2, as well as the signal cascade to the brain.

RA mechanoreceptors are highly sensitive to soft-touch and low-frequency vibration, providing high-acuity tactile feedback that is critical for sensing dynamic aspects of food texture and integrating touch with taste and overall oral sensation^33^. RA responses are typically associated with encapsulated end-organs such as Meissner´s corpuscles and possibly end bulbs of Krause^34^. These structures are located beneath the basal membrane in the lamina propria and are found in regions including the hard and soft palate, fungiform and filiform papillae, gingiva, and cheeks. Meissner's corpuscles exhibit an ellipsoid, lamellated architecture composed of stacked Schwann cells interspersed with Aβ-myelinated nerve endings that branch into unmyelinated fibres, enabling rapid responses to dynamic deformation^35^. Whereas the end bulbs of Krause are spherical corpuscles containing a mix of myelinated and unmyelinated nerve fibres arranged in a convoluted pattern^36^. Besides mechanotransduction, these end organs are equipped with TRP channels, including TRPM8 and TRPV1, suggesting additional roles in nociception and thermosensation. TRPV1 is activated by heat, but also responds to the natural chemical capsaicin, a pungent component of spicy chilies^37^, whereas TRPM8 responds to cold and to compounds such as menthol that evoke a cooling sensation^38,39^.

SA mechanoreceptors comprise Merkel cell–neurite complexes (SAI) and Ruffini endings (SAII)^40^. Merkel cells are highly specialised epithelial cells located in the basal layer of the oral mucosa, particularly in the hard palate and gingiva. In association with Aβ-afferent endings, they form Merkel cell–neurite complexes that are central to sustained touch and fine spatial discrimination. These complexes express high levels of mechanosensitive ion channels, notably PIEZO1 and PIEZO2^41^, which are now considered key molecular transducers of low-threshold mechanosensation. Piezo proteins are large mechanically-gated ion channels that respond directly to membrane deformation^42^. Mechanical stress induces conformational changes that open the channel pore, permitting cation influx and leading to depolarisation and receptor potential generation, which is relayed to adjacent sensory neurons. Although PIEZO2 is widely regarded as the principal transducer of low-threshold touch in somatosensory systems, emerging evidence indicates that both PIEZO1 and PIEZO2 are expressed in distinct oral sensory structures and may differentially contribute to oral mechanosensation. PIEZO2, in particular, is enriched in trigeminal sensory neurons and Merkel cell–neurite complexes, suggesting a role in fine texture discrimination and the perception of surface features during mastication. PIEZO1 has been implicated in higher-threshold, protective mechanosensation in oral tissues, including responses to strong mechanical load^43^. Functionally, Merkeldiskss encode sustained pressure and local indentation, enabling the detection of edges, curvature, and fine surface irregularities that are highly relevant for perceiving graininess, roughness, and particle size during food breakdown^44^. In contrast, Ruffini endings are sensitive to sustained stretch and tissue tension. They are predominantly located in the periodontal ligament surrounding the teeth, where they contribute to the detection of tooth displacement and bite force. Structurally, Ruffini endings consist of dendritic ramifications with expanded axon terminals that interface with collagen fibres and are closely associated with terminal Schwann cells, resembling the organisation observed in Meissner's corpuscles^45^.

Proprioceptors provide information about the position and movement of the tongue and jaw, but also about the magnitude of bite force generated during mastication. By monitoring muscle stretch, joint position, and periodontal ligament tension, they contribute to the fine control of oral motor behaviour and help calibrate chewing dynamics. This sensorimotor feedback can indirectly influence texture perception by shaping bolus manipulation, particle breakdown, and swallowing timing. However, the primary encoding of textural features such as surface irregularity, indentation, and slip is mediated by the low-threshold mechanoreceptors described above^46^.

### Beyond classical mechanoreceptors: polymodal and mechano-chemosensory pathways

Free nerve endings are thin terminal branches of myelinated or unmyelinated sensory fibres that are widely distributed throughout the oral epithelium, including the tongue, lips, palate, cheeks, and gingiva. Unlike encapsulated low-threshold mechanoreceptors, they provide more diffuse but highly salient signals related to mechanical, thermal, and chemesthetic (irritant) stimuli. Their activation is mediated by polymodal ion channels that respond to combinations of membrane deformation, temperature shifts, osmotic stress, and chemical ligands^31^. Members of the TRP channel family are particularly relevant in this context: Beyond TRPM8 and TRPV1, which mediate cooling and heat/capsaicin responses^36–39^, additional TRP subtypes such as TRPA1 and TRPV2-4 contribute to oral sensations associated with pungency, carbonation, weak acids, osmotic changes, and membrane stress^47,48^. Although chemical and thermal activation pathways differ at initiation, they converge at shared gating mechanisms that produce similar cation influx and downstream signalling, explaining why certain chemical stimuli evoke thermal-like percepts.

Some TRP channels, including TRPP2 (PKD2), are responsive to shear stress or fluid flow and may interact with mechanosensitive pathways in epithelial and sensory cells^49^. Importantly, several TRPs can also be sensitised indirectly via G protein–coupled receptor signalling, linking chemical composition to altered mechanical responsiveness^50^.

In addition, proton-sensitive ion channels of the ASIC family and mechanosensitive potassium and sodium channels (such as TREK and ENaC/DEG families) respond to membrane tension or extracellular acidity. In sensory neurons, these channels primarily modulate excitability and shape responses to primary mechanotransducers such as PIEZO channels, rather than acting as independent low-threshold texture detectors. Together, these polymodal pathways broaden the sensory coding of oral stimuli and contribute to the complex integration of mechanical, chemical, and thermal cues that define mouthfeel^51^.

### Multisensory integration of mouthfeel: from oral mechanisms to complex food experiences

All oral mechanoreceptors described above are primarily connected to large-diameter myelinated Aß nerve fibres and follow the classic three-neuron somatosensory pathway (receptor → trigeminal ganglion → thalamus → cortex)^31,52^ as shown in Fig. 2. Peripheral trigeminal afferents from the oral cavity (via V2/V3 branches) enter the pons (middle segment of the brainstem) and terminate in specific trigeminal nuclei (such as the principal and spinal nuclei, including the subnuclei oralis (Sp5O), interpolaris, and caudalis)^53,54^. From there, second-order neurons ascend in the trigeminothalamic tracts (TTT). Proprioceptive and tactile input from the oral cavity has a prominent ipsilateral component via the dorsal region of the TTT (DTTT), whereas most other facial somatosensory input crosses and ascends contralaterally in the ventral region of the TTT (VTTT)^55^. Both pathways mainly terminate in the ventral posteromedial nucleus (VPM) of the thalamus. Third-order neurons project from the VPM to the face/oral region of the primary somatosensory cortex (S1)^56,57^. Trigeminal oral somatosensory input from S1 finally converges with taste and olfactory pathways in the anterior insula and frontal operculum (gustatory cortex), contributing to a unified flavour and oral perceptual experience^58,59^. Because a single bite or sip simultaneously delivers chemical compounds, mechanical forces, and temperature changes, multiple sensory subsystems are activated in parallel, resulting in inherently multisensory mouthfeel perception^58^. The most prominent examples of complex oral experiences are:

#### Carbonation

Carbonation illustrates the convergence of multiple sensory systems. The perception of fizziness does not arise solely from bubble mechanics^60^. CO₂ is converted to carbonic acid at the surface of sour-sensitive taste cells, activating the gustatory system via pH shift detection. Simultaneously, carbonic acid stimulates trigeminal nociceptive pathways, producing tingling and slight burning sensations, while bubble rupture generates localised mechanical pressure. In addition, CO₂-induced chemical irritation activates thermal- and pain-related TRP pathways (e.g., TRPA1). The combined activation of taste, mechanosensory, and chemesthetic systems explains why carbonation is experienced as a complex, dynamic oral event rather than a simple tactile cue^60^.

#### Astringency

Although often described as taste-related, astringency is mechanistically best understood as a tactile phenomenon. It is perceived as drying, rough, or puckering, descriptors that reflect altered mechanical interactions in the oral cavity. The classical mechanism involves interactions between polyphenols (e.g., catechins in tea, cocoa, or wine) and salivary proteins, leading to aggregation of the mucosal pellicle, reduced lubrication, and increased friction at oral surfaces^61^. These changes enhance activation of oral mechanoreceptors during movement, generating the characteristic drying sensation. Emerging evidence suggests an additional, complementary pathway in which astringent compounds may directly interact with mechanoreceptors or alter membrane mechanics, potentially modulating mechanosensitive ion channels^61^. These mechanisms are likely not mutually exclusive and may operate in parallel depending on compound structure, concentration, and food matrix. Because astringency strongly influences food acceptance, understanding its mechanistic basis is critical for the development of plant-based and polyphenol-rich products^62^.

#### Fat perception

A differentiation of fat perception into fat taste and fatty mouthfeel or fat texture perception may be useful. Free fatty acids (FFAs) can be detected chemically via the taste receptors CD36 and GPR120^63^, supporting the concept of a potential 'fat taste'. Some studies indicate that the primary sensing of fat is mediated by FFAs rather than by triacylglycerides^64^ themselves or their associated viscosity^65^. However, human lingual lipase activity releasing non-esterified fatty acids from triacylglycerides appears to be low^63,64,66^, although it may increase with more extensive oral processing^64^. Recently, alternative lipase genes have been identified in human von Ebner’s glands^63^. It has therefore been proposed that naturally occurring non-esterified fatty acids, together with those released by oral lipolysis, may reach concentrations sufficient to elicit a taste response^66^. Consistently, salivary lipolysis and fatty acid sensitivity have been associated with fat perception and preference^64^. Nevertheless, the extent to which these mechanisms contribute to fat perception remains debated. Fat texture, in contrast, is mainly related to the physical properties of fatty foods, such as creaminess, viscosity, and lubrication, which are detected by mechanoreceptors and processed through somatosensory pathways^67,68^. In particular, tribological cues may play an important role in the perception of fatty mouthfeel. Investigations at the neuronal level in primates found that fat-responsive neurons responded not only to oils but also to non-fat stimuli, producing similar textural characteristics and lubrication properties^69^. Their activity was correlated with the coefficient of sliding friction, a measure related to oral lubrication^70^, but not with apparent viscosity. These neurons were largely distinct from neurons responding to fatty acids, suggesting that fat texture can be perceived independently of oral gustatory inputs mediated by fatty acids^69^. Tribological cues could therefore provide a distinct component of fat-related sensory perception^70^. Moreover, neuronal activity has been associated with pleasantness ratings of fat texture and taste^70^, supporting a potential contribution of oral texture perception to the hedonic perception of fatty foods. In addition, volatile products from lipid breakdown (e.g., aldehydes) contribute retronasally to flavour enhancement^68^. Thus, fat perception arises from the integration of chemical detection, lubrication-dependent mechanosensation, and olfactory input.

### Crossmodal interactions in flavour perception

Crossmodal interactions are central to the unified perception of flavour, reflecting integration across tactile, thermal, chemical, olfactory, auditory, and visual modalities. These interactions can occur at the perceptual level or involve higher-level cognitive and emotionally mediated processes^71^.

#### Texture-taste

Texture significantly modulates taste perception and vice versa. Beyond surface roughness affecting perception, softness and viscosity influence perceived sweetness and overall flavour intensity^72^. Soft textures can enhance sweetness perception and sweetness in turn reinforces perceived softness^73^. Importantly, sweetness does not necessarily increase viscosity but interacts perceptually with texture and aroma to enhance richness and indulgence, particularly in fat-containing products^2^. Sugars and fats can also mask astringency and roughness, increasing perceived smoothness and thickness on the palate^74^.

#### Texture-smell

Aroma can also modulate perceived texture. Vanilla provides a well-established example: Beyond enhancing sweetness, vanilla aroma increases perceived creaminess and thickness, particularly in dairy-based systems^2^. When congruent with fat-derived sensory cues, retronasal vanilla aroma amplifies the impression of body and smoothness, even without measurable changes in rheological properties. This demonstrates that texture perception is not determined solely by mechanical input but is shaped by central integration of olfactory and somatosensory signals.

#### Texture-sound

Carbonation in beverages is one example illustrating that auditory cues contribute to expectations of freshness^75,76^, but the in-mouth perception of fizziness is dominated by trigeminal and somatosensory activation, with visual and auditory signals modulating intensity rather than replacing oral input^71^. In terms of crunchy or crispy foods, the auditory input only becomes dominant in the mastication and swallowing process^77^, determining the pleasantness of fruits, vegetables, or dried foods being provided by tactile, mechanical, and acoustical enjoyment, hence driving altogether freshness evaluation and liking^76^.

#### Texture–vision

Vision can actively shape how rough, smooth, creamy, crunchy, or thick something feels in the mouth by generating predictions about the mechanical properties of a given food, thereby biasing the interpretation of subsequent oral tactile signals^78^. For example, a glossy, homogeneous surface may appear creamier and thicker, whereas sharp edges and matte or dry-looking surfaces that visibly fracture can indicate crunchiness and crispness. Colour also matters: red and blue colours have been shown to increase perceived creaminess and crunchiness in snack foods, even when the physical texture is unchanged^79^. Another study showed that yellower butter was judged as more spreadable, a texture-related property, than paler butter under normal lighting; however, this effect disappeared under red light, indicating that the visual colour cue was decisive for the texture judgement^80^. In addition to colour, illumination itself can be a powerful visual modulator. In a study using 3D surfaces, changing the direction of illumination and thereby the position of shadows and highlights that enhance perceived surface roughness, was sufficient to bias participants toward incorrect haptic texture judgements^81^. This demonstrates that the visual appearance of food can establish strong priors about mouthfeel and texture, which are then integrated with oral signals within a predictive sensory framework. This further illustrates that texture perception is embedded within broader multisensory integration processes^82^.

## Sensory descriptors and measuring mouthfeel

**Fig. 3:**
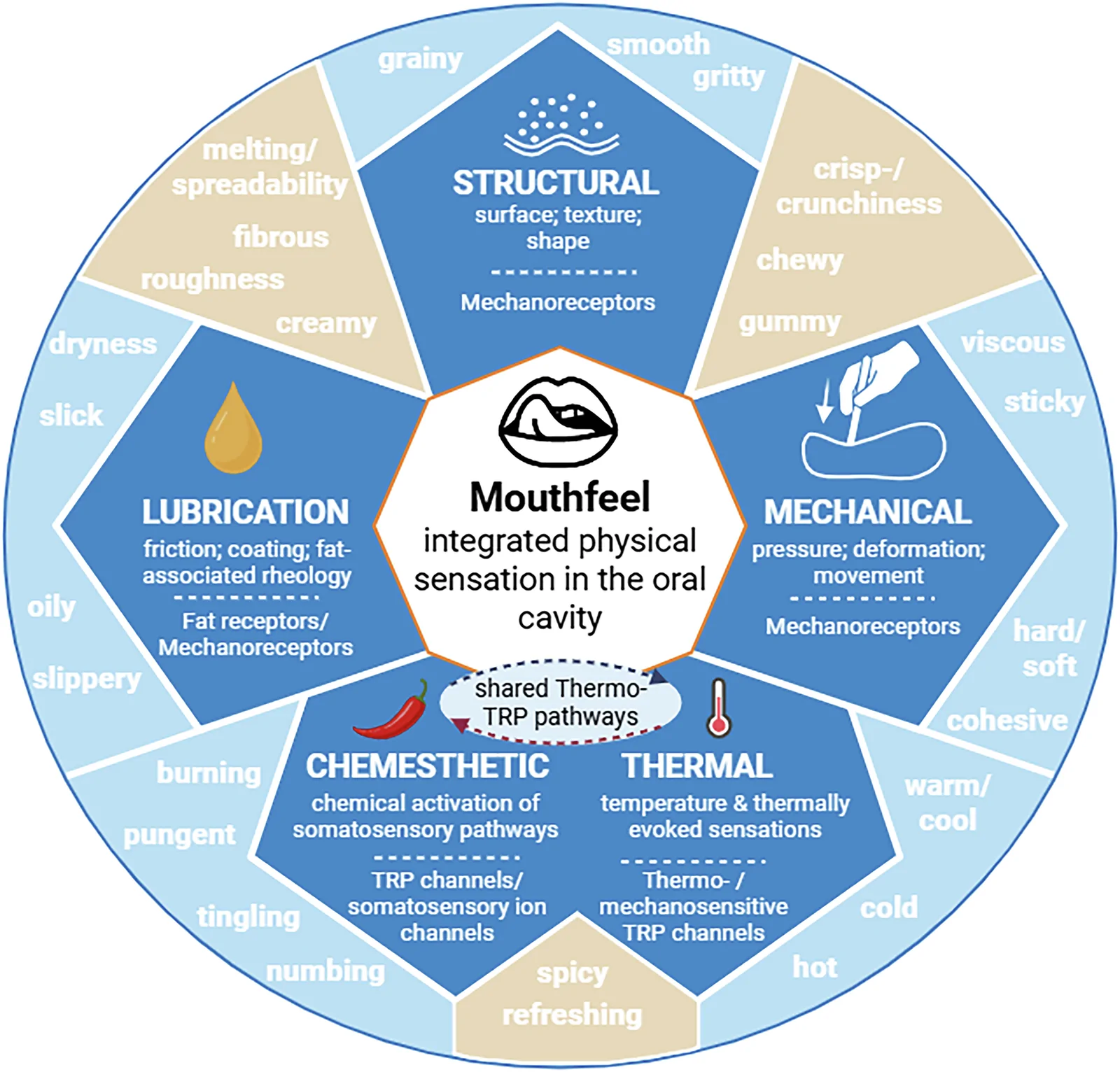
Mouthfeel descriptors categorised by the type of stimulus. Since chemesthetic and thermal stimuli share similar thermo-TRP pathways, they are grouped into the same main category, with distinct descriptors. Other than that, the descriptors (light blue) are categorised in lubrication, structural, and mechanical stimuli (dark blue). Overlapping descriptors between categories are highlighted in beige. Created in BioRender. https://BioRender.com/71z349n.

Combining texture sensations with cross-category classifications, Klosse^83^ introduced a general mouthfeel-based flavour model consisting of the triad 'contracting/drying', 'coating/filmy', and 'flavour richness'. Although this influential model builds on a long history of mouthfeel and texture descriptions^4,7^, it simplifies an extremely complex sensory experience into a limited number of categories that may not capture all relevant tactile or chemesthetic sensations across different product types. In addition, terminology in this area is not standardised across the broader sensory science community, making consistent wording one of the main challenges when describing mouthfeel^2^. In literature, numerous terms are used to describe mouthfeel and texture as multi-parameter sensory attributes. These are often organised into sensory wheels or lexicons, which serve as useful communication tools among diverse audiences such as sensory panellists, product developers, marketers, and suppliers. However, these tools also have several limitations^84,85^. They typically reflect expert language and are often developed for a specific product category, making them difficult to transfer to other foods or mixed dishes. For some products, multiple lexicons with different terminology exist, which may cause confusion, while for many others no standardised lexicon is available^86^. For a more detailed overview, Agorastos et al. ^87^ summarised the main attribute groups of existing mouthfeel wheels and sensory lexicons across food categories. Their work highlights substantial differences in attribute classifications, even within the same product group. Since many existing wheels require further refinement, Fig. 3 proposes a simplified, exemplary framework that categorises four potential descriptor groups—mechanical, structural, lubrication, and chemesthetic & thermal—based on the type of stimulus and the receptor systems involved. This visualisation illustrates both the diversity of mouthfeel sensations, still with mechanoreceptors involved in every category, and the considerable overlap between descriptor groups.

Another important aspect is the cultural variation in how mouthfeel is described. For example, the Japanese language contains more than 400 expressions for describing oral sensations during food consumption, whereas American English includes only around 80 such descriptors^88^. This difference reflects both linguistic diversity and the remarkable sensitivity of the human sensory system in capturing complex, cross-modal mouthfeel experiences. Instrumental techniques such as texture profile analysis (TPA), rheometry (captures bulk flow and deformation properties), and tribometry (captures friction and lubrication between oral surfaces) provide objective physical measurements of mouthfeel-related properties, but they cannot fully capture the dynamic, perceptual, and biological aspects of oral sensation^89^. In contrast, sensory panels and consumer tests measure actual human perception and liking^5^. Attributes such as 'body', 'richness', or 'drying', which arise from multiple simultaneous sensory inputs, therefore cannot be reliably predicted from single rheological or structural parameters alone. Consequently, understanding mouthfeel requires a multimodal approach that integrates (bio)physical measurements^90^, biochemical characterisation (for example compound-receptor interactions or saliva composition), and rigorous sensory evaluation. Such integrative strategies are essential for building predictive frameworks that link food structure, oral perception, and consumer experience^16,91^.

These perceptual and methodological considerations also highlight why mouthfeel plays a critical role in eating behaviour and dietary choices, which will be explored in the following section.

## How mouthfeel influences eating and health

**Fig. 4:**
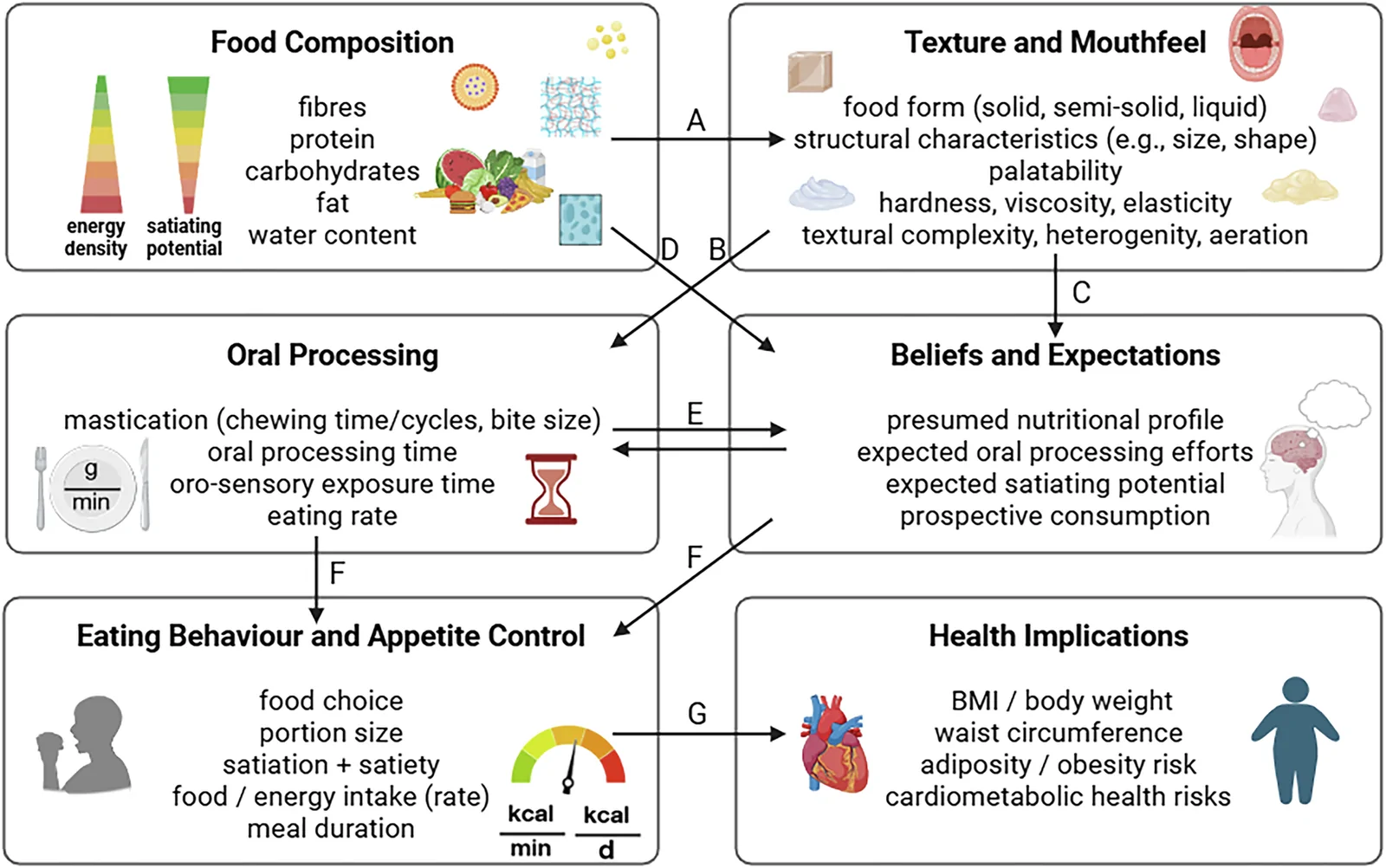
Connections between food composition, perceived texture and mouthfeel, oral processing, beliefs and expectations about consumed foods, eating behaviour and appetite control, and their potential implications for overall health. Food composition, structure, and processing determine physical texture and its perception in the oral cavity (A)^22,77,126,129,130^. Perceived food form, structural characteristics, and mouthfeel properties, such as hardness, viscosity, and elasticity, influence mastication effort, oral processing time, oro-sensory exposure, and eating rate (B)^20,21,119–122,126,147,148,151,152,157^. Texture and mouthfeel may also shape beliefs and expectations regarding a food’s nutritional profile, the effort required for oral processing, its satiating potential, and prospective consumption (C)^12,22,119,129,145–147,157^. Initial expectations are formed before consumption based on visual, olfactory, contextual, and prior-experience cues (D)^22,129,145,146^. These expectations may guide initial oral processing behaviour, which is subsequently adjusted in response to sensory feedback during consumption. At the same time, sensory experiences during oral processing can update beliefs and expectations about the food (E)^22,145,147,153^. Together, oral processing and related cognitive and sensory processes influence eating behaviour, satiation, and appetite control (F)^20–22,77,79,92,118–122,126,135,147,148,151,153,158^, with potential implications for energy balance and longer-term health (G)^24,92,143,149,150,158^. For example, liquids and soft or thin-textured foods that require little oral processing can facilitate rapid consumption and limit oro-sensory exposure. Particularly when such foods are energy-dense and highly palatable, this may contribute to greater energy intake and weaker satiation which, if sustained over time, may promote weight gain or increase cardiometabolic risk. Conversely, solid, hard, viscous, or elastic foods that require more oral processing may increase expected satiation, prolong oro-sensory exposure, and reduce eating rate, thereby supporting satiation and potentially lowering energy intake. Created in BioRender. https://BioRender.com/71z349n.

The texture of foods and its oral perception, i.e., mouthfeel, as an integral part of flavour, has profound implications for consumers’ food choice and eating behaviour^12–15,21^. Although often underrated and unnoticed by consumers unless perceived as unpleasant or inappropriate, mouthfeel exerts a substantial influence on dietary intake and nutritional patterns^14,20,22^. Its effects extend beyond momentary sensory experience and can shape eating rate, portion size, and ultimately health outcomes (Fig. 4)^12,20,21,92^. In this section, we highlight the multiple relationships between food texture, mouthfeel perception, eating behaviour, and their broader implications for health.

### Mouthfeel as a key driver in food choice and food rejection

Textural characteristics and the associated mouthfeel are major determinants of perceived food quality and consumer acceptance^93,94^ across all age groups^13^. Preferences for texture are highly food-dependent^11,15^ and rarely based on a single attribute. Instead, they often reflect combinations of textural features (e.g., creaminess), dynamic changes during oral processing (e.g. breakdown of bread), or contrasts within a product (e.g., juiciness and firmness in fruits)^15,77,95–97^. In many cases, perceived texture can be as important as—if not more important than—aroma or taste and is closely linked to emotional responses^98–101^. This connection is supported by interactions between sensory systems, including fat- and viscosity-sensitive neurons, and brain networks involved in memory, emotion, and reward, which together shape pleasure, motivation, and food choice^12,28,102–104^. While taste and odour are commonly cited as drivers of liking, consumers frequently refer to texture when explaining food rejection^11,15,93,96^. Textures perceived as unexpected, inappropriate, or inconsistent with prior expectations are particularly associated with rejection^4,7,12^. The ability to reject foods based on textural cues serves as an important protective function linked to negative emotional reactions by supporting safety and well-being (e.g., signs of spoilage) as well as culturally shaped beliefs or individual preferences^15,28,100^. This is especially relevant for vulnerable populations with specific dietary needs, such as older adults or individuals with dysphagia, xerostomia, or other conditions that limit oral processing, swallowing, and digestion^105^.

However, texture and mouthfeel can also contribute to restrictive or selective eating patterns in both children^106–108^ and adults^14^ with substantial unfavourable outcomes for nutritional quality and general health^109–111^. Picky eating is frequently directed toward low-energy, minimally processed foods such as vegetables, fruits, and meats^15,109,110^, potentially compromising overall diet quality and increasing reliance on energy-dense alternatives^23,109,110^. In severe cases, this may contribute to increased body weight^23,111^ and can even lead to malnutrition in children and adults^109,110,112^. Individuals requiring texture-modified diets, including those with dysphagia or other medical conditions, are similarly at risk, as modifications can reduce sensory enjoyment and dilute nutrient density, resulting in lower energy and protein intake^105,113–115^. Consequently, mouthfeel and texture become increasingly important across the lifespan^13^. In patients experiencing taste or smell loss following respiratory infections such as COVID-19, mouthfeel can partly compensate for reduced sensory input^116^, although altered oral sensations may also negatively affect quality of life^117^.

### Mouthfeel as a modulator of eating behaviour, satiety, and metabolic health

Food texture and mouthfeel play a crucial yet often underestimated role in healthy eating and appetite regulation^21^. Food selection based on palatability and (dis-)liking, energy density, oral processing dynamics, and subsequent effects on satiation and satiety are strongly shaped by textural properties and mouthfeel sensations^118^ (Fig. 4). Texture and mouthfeel exert both direct and indirect effects on oral processing properties by influencing bite size, eating rate, and oro-sensory exposure time^20,119,120^, with downstream consequences for satiation (intra-meal satiety or fullness during eating) and satiety (post-ingestive satiety or suppression of hunger between meals)^93^. These effects are observed in more solid-textured foods (harder), semi-solids, or liquids with higher viscosity or creaminess, in chewy, elastic, springy, cohesive, mealy, sticky, or thick foods^21,118,119,121–123^. In this way, texture and mouthfeel may modulate eating rate, shaping energy intake, and influencing metabolic health (Fig. 4).

Texture perception (i.e., mouthfeel) also contributes to palatability^124^, describing pleasantness, desire-to-eat and hedonic reward evoked during oral exposure^125^. Energy-dense foods such as those rich in fat often possess textural properties and mouthfeel characteristics that enhance palatability^126,127^ leading to a reward response and facilitating faster consumption due to reduced oral processing effort^21^ (Fig. 4). From an evolutionary perspective, preference for such high-energy textures may have supported survival under conditions of scarcity^128^. Nowadays, in contemporary food environments characterised by abundance, however, these properties may promote rapid consumption with limited oro-sensory exposure, reducing perceived satiation potential despite high caloric density^77,129–131^. While similar patterns are observed for carbohydrate-rich foods, proteins and fibres (typically lower in energy density) are associated with enhanced oral processing and prolonged oro-sensory exposure, contributing to greater satiation and satiety^77,129–131^ (Fig. 4). Although mouthfeel can cue aspects of a food’s nutritional profile through its influence on oral processing, humans cannot reliably detect energy density^126^. This mismatch contributes to disproportionate intake of highly palatable, energy-dense foods^132,133^, potentially increasing energy consumption without proportional satiety^134^ and hence, increasing overall food intake^121^. Over time, such patterns may promote overconsumption^135,136^, eating disorders and obesity^137^, and disturbances in healthy appetite regulation^138–141^, including reward-related eating behaviours^104,142,143^ that affect nutritional and mental health^136,144^.

Importantly, texture and mouthfeel can shape expectations of fullness independently of nutrient composition. Properties such as thickness, viscosity, or creaminess may signal higher satiating potential through learned associations between oral cues and post-ingestive outcomes^22,145,146^. These cognitive and sensory mechanisms operate alongside physiological signals, jointly influencing food choice, portion size, and intake^12,21^. Notably, texture-driven reductions in energy intake are often reported without subsequent compensatory overeating^121,147^. Meta-analyses by Stribiţcaia et al. (2020)^119^ and Appleton et al. (2021)^120^ provide comprehensive evaluations of the evidence linking texture and mouthfeel to appetite regulation and pleasantness. In summary, physical form (solid, semi-solid, and liquid) and viscosity can modulate appetite ratings without diminishing hedonic value. Other mouthfeel attributes such as hardness, elasticity, chewiness, thickness, creaminess, or stickiness are consistently associated with slower eating rates and enhanced satiation. Some studies further suggest that food texture and mouthfeel may influence gastro-intestinal peptide release and hormonal satiety signalling^122,123^, although evidence remains inconsistent and the magnitude of these effects is debated.

Overall, these texture- and mouthfeel-driven modifications in eating behaviour may contribute to lower daily food and energy intake^148^ and reduced risk of overeating, weight gain^149^, elevated body mass index (BMI), excess adiposity^150^, larger waist circumference, obesity, and cardiometabolic diseases^24,92^ (Fig. 4). Texture and mouthfeel therefore represent not only potential risk factors but also modifiable and accessible levers for enhancing oral processing, moderating intake, steering appetite control, supporting metabolic health, and facilitating healthy eating^126^.

### Engineering texture for healthier eating

Beyond ingredient substitution, texture modification represents a powerful tool to steer and support healthier eating behaviour. Slowing eating rate and prolonging mastication, thereby extending oro-sensory exposure time, have been shown to enhance both satiation and satiety^151–153^. Such effects can be achieved by modifying foods toward harder, firmer, thicker, or more elastic textures with reduced lubrication^119,120,126,152^. Even modest changes in texture can measurably influence eating behaviour^23^, importantly without diminishing pleasantness or overall appeal^21,120^. Several formulation strategies can enhance perceived thickness and modulate oral processing. These include reducing emulsion droplet size^129^, restructuring emulsions^154^, or incorporating hydrocolloids (e.g., xanthan gum, pectin, and carrageenan) to increase viscosity and perceived thickness^105^. Enhancing the content of beneficial fibres and proteins offers a combined approach for improving nutritional quality and altering oral processing dynamics. Textural attributes such as elasticity, chewiness, or springiness can prolong mastication and enhance satiation, with subsequent effects on satiety^155^. However, fibres and proteins exert heterogeneous effects on texture and intake, which is why their impact must be considered individually^120^. Yet, modifications in food texture aimed at stimulating oral processing do face limitations. This is particularly relevant for fat-related food products, where the perception of fat texture and the associated reward response are correlated with lubricity^70,156^. Extensive alteration of food properties may therefore reduce liked textural characteristics and result in unintended compensatory responses^156^. Aeration represents another promising strategy. Incorporating air increases food volume while lowering energy density, thereby enhancing fullness and delaying subsequent consumption^119,120,152,157^. Designing foods with more complex and dynamic textural profiles, eliciting multiple sensations during oral processing, may further enhance receptor stimulation and promote earlier satiation^118,119,147^. This phenomenon has been linked to sensory fatigue, potentially contributing to reduced intake and prolonged satiety^147^.

Collectively, these approaches demonstrate that texture and mouthfeel can be strategically leveraged in food product design and dietary planning to promote appetite control and healthier consumption patterns. Given the strong influence of texture perception on intake and metabolic health, texture-based reformulation offers a practical avenue to address overconsumption, obesity, malnutrition, and diet-related chronic diseases^62^. Importantly, advancing such strategies requires not only technological innovation but also a deeper understanding of the physiological mechanisms underlying mechanosensation and oral texture perception. Integrating mechanistic insight with food design will be essential to harness mouthfeel as a scientifically grounded lever for public health. In the following section, we discuss approaches of textural design to face the nutritional challenges.

#### Potential of fat replacers in low-fat products

**Fig. 5:**
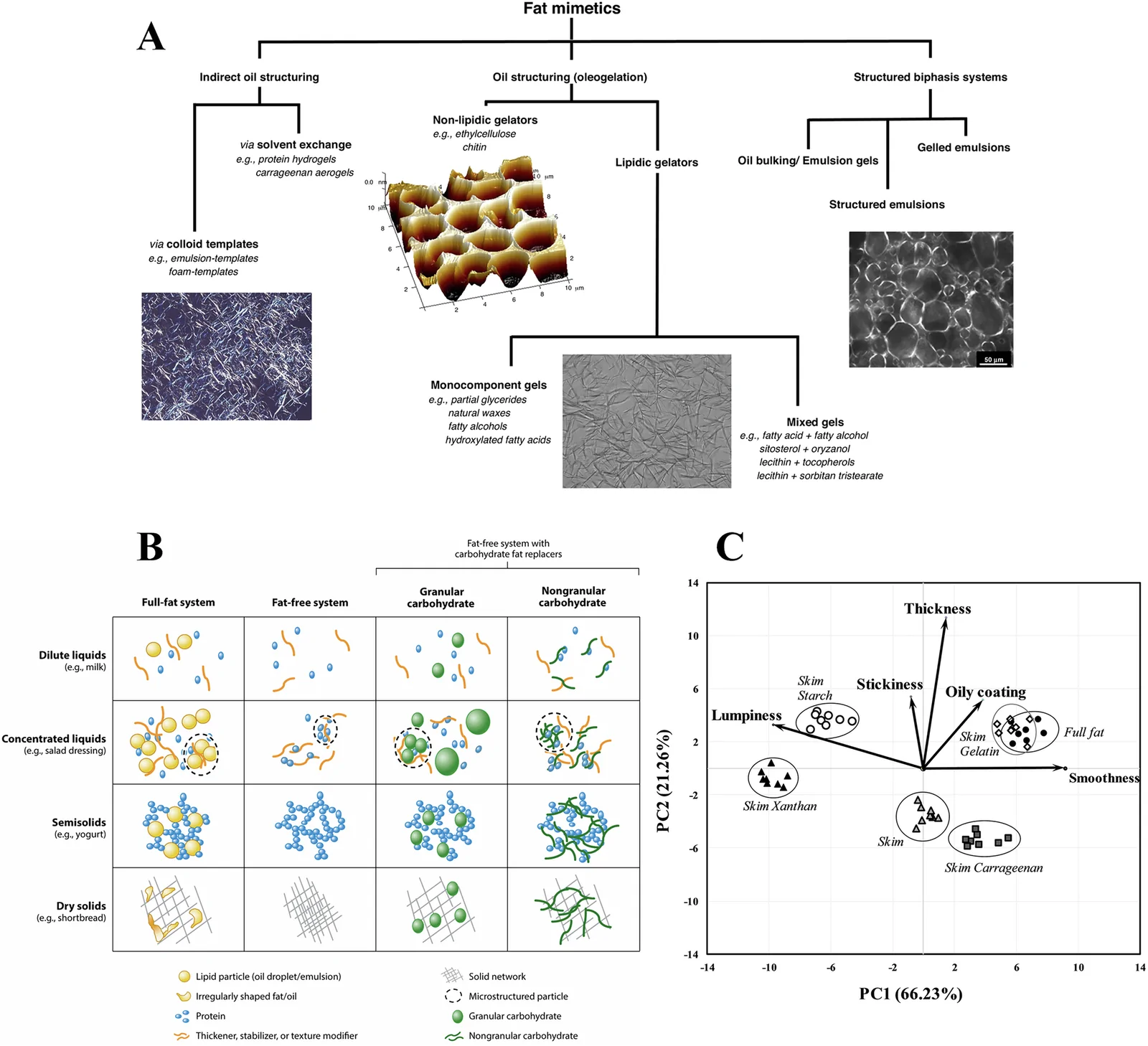
Recent advances and applications of fat replacers in literature. **A** Strategies of designing fat mimetics with exemplary microstructural images of different samples. Illustration adapted from ref. ^161^. **B** Structural characteristics of full-fat and fat-free systems in different physical states or products compared to carbohydrate-based fat replacers in the fat-free systems. Image adapted from ref. ^160^. **C** Principal component analysis on sensory ratings of low-fat yoghurt when using various hydrocolloids as fat replacers highlighting the individual contribution of different hydrocolloids to textural and mouthfeel characteristics. Figure adapted from ref. ^197^.

Appealing sensory properties such as creaminess and thickness are strongly linked to fat content^67,129,158^. However, high-fat textures are also associated with high energy density, low satiating efficiency, and enhanced palatability, contributing to adverse health outcomes^159^. Consequently, considerable effort has focused on replacing fats with functional ingredients that stabilise, emulsify, gel, or thicken while preserving textural characteristics, lubrication behaviour, and desirable mouthfeel. Fat replacers derived from carbohydrates^160^ (Fig. 5B), proteins, or lipids can partially or fully substitute fats^161^ (Fig. 5A) while maintaining acceptance, palatability, and emotional response^158,160,162^ (Fig. 5C). Comprehensive overviews of available replacers are provided elsewhere^159^. When appropriately formulated (Fig. 5C), such products can reduce fat and caloric intake and support weight management, potentially lowering risks of diabetes, hypertension, and cardiovascular disease^159,160^. Nevertheless, fat substitution does not justify unrestricted consumption, as excessive intake or high levels of replacers may carry unintended effects^159^. Overall, modifying fat-driven texture remains a powerful strategy for improving dietary quality without compromising sensory appeal.

#### Innovations in plant-based products

**Fig. 6:**
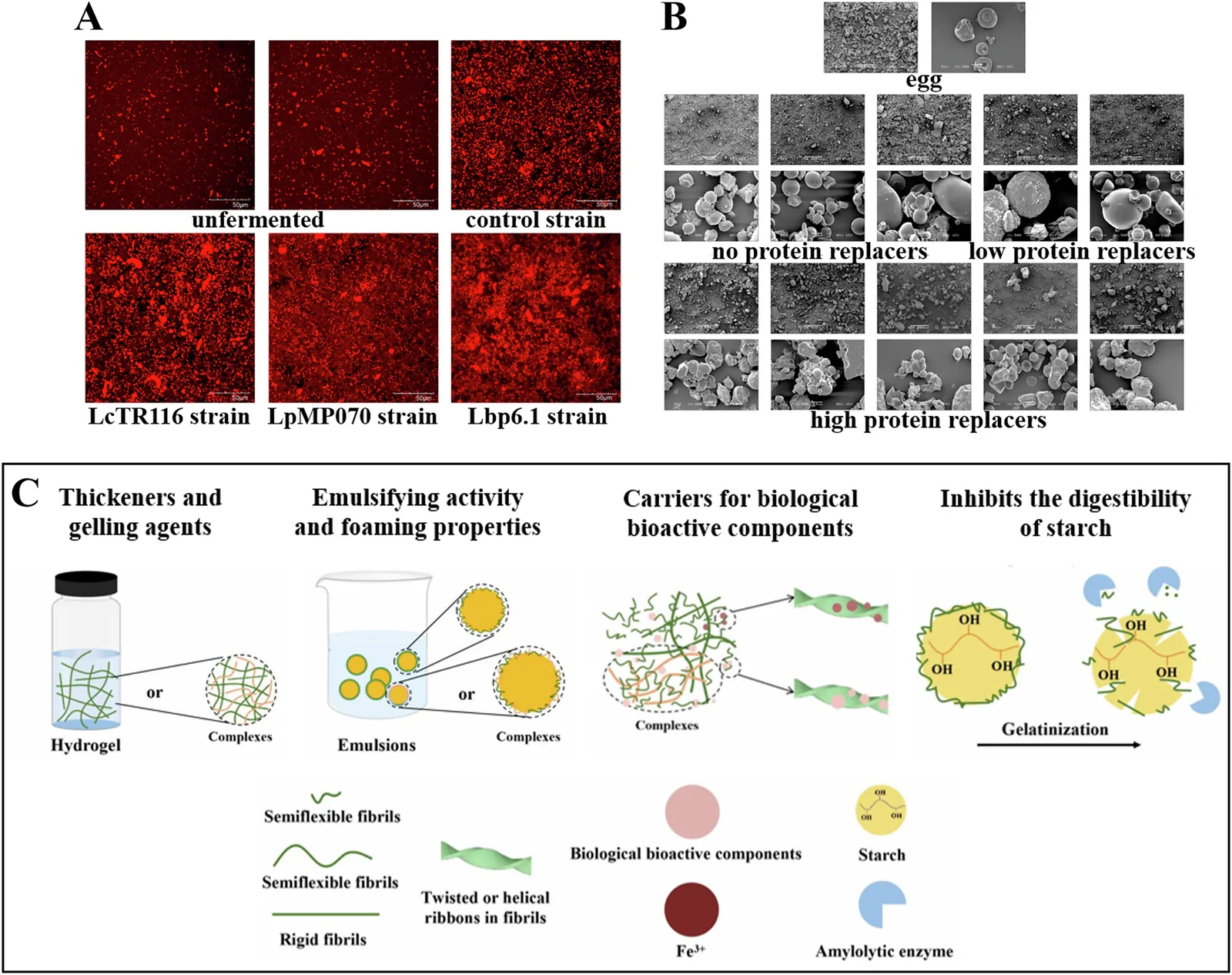
Recent advances and applications of plant-based alternatives in literature. **A** Microstructure of unfermented and fermented lentil protein gels as yoghurt alternatives after Nile blue staining and visualisation with confocal laser scanning microscopy. Images adapted from ref. ^169^. **B** Scanning electron micrographs of pound cake batter with egg or egg replacers (x50 and x1500). Images adapted from ref. ^168^. **C** Overview of potential applications of plant-based amyloid-like nanofibrils in food products. Illustration adapted from ref. ^170^.

Reducing consumption of animal-derived products has the potential to improve health and wellbeing^163^. Plant-based alternatives typically contain less fat and cholesterol while having a higher fibre content^164^, contributing to reduced risks of cardiovascular disease and mortality^165^. However, nutritional limitations, particularly with respect to protein quality^164^, remain a concern. From a sensory perspective, plant-based analogues often fail to replicate the desired texture and mouthfeel of animal-derived products^162^, despite texture being a key driver of acceptance^166,167^. Figure 6B shows one example of commercially available plant-based egg-replacers in pound cake being inferior—although having comparable textural values—regarding appearance, physical and textural properties and nutritional value^168^.

Strategies to improve texture include hydrocolloid and gelling systems, material blending, enzymatic modification, homogenisation, nanoencapsulation, and fermentation techniques^165,167^. Current research therefore focuses on balancing taste and nutritional value with desirable mouthfeel, ensuring that health-promoting plant-based options are both acceptable and satisfying^168,169^. For example, exploring different strains of bacteria for fermenting gels based on lentil protein isolate may yield sugar-reduced lentil yogurt alternatives or achieve high acceptance, good texture ratings, and yoghurt-like flavour (Fig. 6A)^169^. Moreover, recent studies highlight the potential of self-assembled amyloid-like nanofibrils in plant-based foods as texture enhancers or modifiers, carriers for bioactive compounds or starch digestion inhibitors for low-sugar or low-calorie products (Fig. 6C)^170^.

#### Innovations in medical nutrition products

**Fig. 7:**
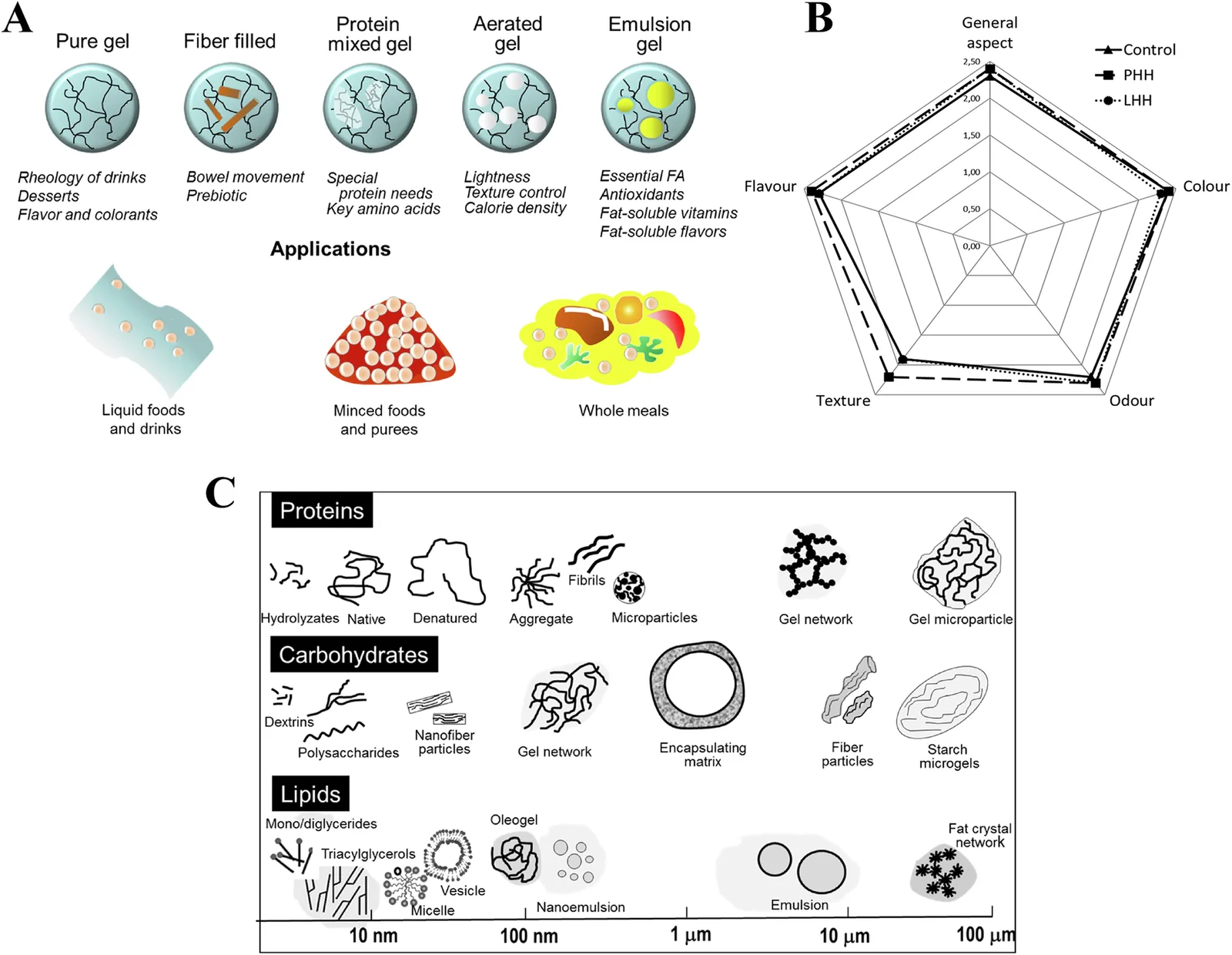
Recent advances and applications of texture-modified foods for vulnerable populations in literature. **A** Range of microgels and their application in a variety of food products, especially texture-modified foods for vulnerable populations. Overview adapted from ref. ^198^. **B** Sensory ratings of canned mackerel pâté as a high-quality food preparation for the elderly population fortified with liquid hydrolysate of sea cucumber *H.forskali* (LHH) or powder hydrolysate holothurians (PHH) in relation to the non-fortified control. Figure adapted from ref. ^180^. **C** Relevant molecules and structural elements in the design of soft textured modified foods for the elderly and specific dietary needs sorted by size. Schematic adapted from ref. ^198^.

Vulnerable groups, including older adults and individuals with disease-related impairments, have specific textural requirements^106,117,171^. Texture-modified foods are essential for safe intake in conditions such as dysphagia, yet product availability and nutritional quality are often limited^105^. Progress in fortification and enrichment combined with targeted textural design is therefore strongly advocated^105^. Adapting food structure to meet functional needs can improve energy and nutrient intake while enhancing sensory appeal^105,115,172^. Although softer textures facilitate intake, modifications may compromise sensory quality and reduce acceptance^115^. Therefore, techniques that preserve both nutritional and sensory integrity, such as blade tenderisation^173^, enzymatic impregnation^174^, freeze-thawing^175,176^, aeration^177^, or the use of natural polymer microgels^105,178,179^ (Fig. 7A, C), offer promising solutions. An additional approach is fortification of foods, for example with sea cucumber hydrolysates that have good nutritional properties (minerals, essential amino acids, antioxidants, anti-hypertensive, and anti-inflammatory), while achieving similar sensory qualities or even improving texture (Fig. 7B)^180^. Another method for improving the palatability, pleasantness, and satisfaction associated with texture-modified foods involves modifying auditory input using pseudo-chewing sounds that mimic crunchy textures^181^. This has been shown to alter the perception of softened foods, making them seem stiffer, rougher, and more complex in texture, thereby exploiting the connection between auditory feedback and oral processing to enhance eating pleasure.

Since texture and mouthfeel influence dietary behaviour across the lifespan^13^, advances in texture design are essential to ensure both safety and sensory satisfaction in diverse populations.

Beyond established reformulation strategies, emerging technologies and interdisciplinary approaches offer new opportunities to deepen mechanistic understanding of mouthfeel and to address nutritional challenges with greater precision, potentially even enabling personalised texture design.

### Future directions: from mechanisms to precision texture design

Much of our current understanding of mechanosensation derives from studies of cutaneous sensory neurons^40^. However, oral mechanosensation differs fundamentally, as signals from the oral cavity are processed via trigeminal ganglion neurons rather than dorsal root ganglia^31^. Advancing fundamental research on trigeminal sensory pathways is therefore essential to fully understand the mechanisms underlying mouthfeel and flavour perception.

Neurophysiological studies in animal models have begun to characterise trigeminal mechanosensory neurons based on conduction velocity and adaptation kinetics, revealing functional diversity in how touch, pressure, and movement are encoded across oral tissues^52^. At the same time, emerging biophysical approaches provide new insight into the cellular basis of oral mechanosensation. Biophysics play a major role in understanding the physical aspects of mouthfeel and complementary methodologies including rheology and tribology remain central in quantifying deformation, friction, and lubrication properties of foods during oral processing^5^. Techniques such as atomic force microscopy (AFM) allow investigation of mechanosensitive receptor activation, membrane mechanics, and interactions between food-derived modulators and sensory cells^90,182^. Together, these approaches bridge molecular mechanotransduction with macroscopic texture perception. Beyond physiology, interdisciplinary fields are expanding our understanding of flavour perception. Neurogastronomy and psychophysics are emerging trends that integrate neuroscience, gastronomy, and consumer research to explore how the brain processes complex sensory experiences^183–185^. Such insights may create more effective strategies for designing foods that balance pleasure with health.

Computational approaches, particularly artificial intelligence (AI), are increasingly applied to flavour and texture prediction^186,187^. Machine-learning models trained on molecular, rheological, and sensory datasets can predict how ingredient substitutions affect attributes such as elasticity, cohesiveness, and mouthfeel. AI-driven formulation may accelerate the identification of novel flavour compounds, optimise texture profiles, and support personalised nutrition strategies. However, reliance on in silico models carries limitations, including dataset bias, safety assessment challenges and potential allergenicity concerns^188–190^. Integrating computational tools with experimental validation remains essential^186,187^. Similarly, electronic tongues and noses offer powerful tools to characterise taste, odour, and composition of foods by combining multisensor arrays with advanced signal processing and machine learning^191^. These systems are increasingly used for quality control and product characterisation, providing rapid, non-destructive, and scalable analyses while reducing reliance on human sensory panels^192^. However, limitations remain, including high costs, lack of standardisation, susceptibility to interferences, and the need to validate outputs against human perception. With ongoing advances in sensor technology and data analysis, these approaches are expected to expand their applicability in food research and development^193^.

Emerging food technologies further enlarge possibilities for precision texture design. Three-dimensional (3D) food printing enables spatial structuring of ingredients with distinct rheological and compositional properties, allowing fine control over sensory attributes, including mouthfeel^194^. Such approaches are particularly promising in healthcare and elderly nutrition, where tailored textures can improve safety and acceptance^195^. Although there is a wide variety of materials employable in 3D-food printing, material constraints, consumer acceptance, stability issues, and regulatory considerations remain challenges.

Finally, the growing evidence linking food structure, mouthfeel, and health highlights the need to move beyond nutrient-centric dietary frameworks. Current nutrition policies primarily focus on composition (e.g., macronutrient content), while largely neglecting structural and textural properties. Incorporating parameters such as physical form, particle size, viscosity, and processing level into dietary guidance may strengthen public health strategies^62,196^. A more integrated perspective that combines physiology, food structure, and behavioural science will be critical to leverage mouthfeel as a scientifically grounded strategy for improving diet quality and health outcomes.

## Conclusion

It is well established that food enjoyment is closely tied to quality of life, and this review has highlighted how texture and mouthfeel are powerful determinants of flavour perception, eating behaviour, and health. Mouthfeel represents a central pillar of flavour, emerging from the integration of chemosensory and somatosensory signals in the oral cavity and involving a diverse array of receptors and neural pathways. Despite its importance, the mechanisms underlying mouthfeel perception remain incompletely understood, contributing to challenges in classification, measurement, and targeted design. The research reviewed here underscores the inherently multisensory nature of mouthfeel, arising from dynamic interactions among food structure, saliva, oral receptors, and central neural processing. Texture perception does not act in isolation but interacts with taste, aroma, vision, and prior experience to shape overall food perception and acceptance. These complex interactions must be considered in global food design, where sensory satisfaction, nutritional quality, sustainability, and cultural context converge.

Texture and mouthfeel influence dietary patterns in both beneficial and adverse ways. Aversions to certain textures can promote restrictive or selective eating with negative nutritional consequences, while highly palatable textures may enhance reward responses and drive overconsumption. At the same time, strategic modulation of texture offers opportunities to support appetite control and healthier eating behaviours without compromising enjoyment.

Although mouthfeel remains more difficult to quantify than taste or aroma, advances in empirical, physiological, biophysical, and computational methodologies are beginning to close this gap. A deeper mechanistic understanding of oral mechanosensation, integrated with sensory science, food technology, and behavioural research, will be essential to utilise texture as a scientifically grounded lever for improving diet quality and metabolic health. Continued interdisciplinary research is needed to clarify how mouthfeel shapes consumer preference, energy intake, satiation, satiety, and long-term metabolic outcomes.

## Data Availability

No datasets were generated or analysed during the current study.

## References

[1] Taylor, A. & Linforth, R. Flavour release in the mouth. *Trends Food Sci. Technol.* **7**, 444–448 (1996).

[2] Wolinska-Kennard, K., Schönberger, C., Fenton, A. & Sahin, A. W. Mouthfeel of food and beverages: a comprehensive review of physiology, biochemistry, and key sensory compounds. *Compr. Rev. Food Sci. Food Saf.* **24**, e70223 (2025). 10.1111/1541-4337.70223 PMC12235588 40625310

[3] Delwiche, J. The impact of perceptual interactions on perceived flavor. *Food Qual. Prefer.* **15**, 137–146 (2004).

[4] Szczesniak, A. S. Texture is a sensory property. *J. Food Qual. Prefer.* **13**, 215–225 (2002).

[5] Stokes, J. R., Boehm, M. W. & Baier, S. K. Oral processing, texture and mouthfeel: from rheology to tribology and beyond. *Curr. Opin. Colloid Interface Sci.* **18**, 349–359 (2013).

[6] Gawel, R., Smith, P. A., Cicerale, S. & Keast, R. The mouthfeel of white wine. *Crit. Rev. Food Sci. Nutr.* **58**, 2939–2956 (2018). 10.1080/10408398.2017.1346584 28678530

[7] Guinard, J.-X. & Mazzucchelli, R. The sensory perception of texture and mouthfeel. *Trends Food Sci. Technol.* **7**, 213–219 (1996).

[8] Bourne, M. C. in *Food Texture and Viscosity* Ch 1 (Academic Press, 1982).

[9] Foegeding, E. A., Vinyard, C. J., Essick, G., Guest, S. & Campbell, C. Transforming structural breakdown into sensory perception of texture. *J. Texture Stud.* **46**, 152–170 (2015).

[10] Peyrot des Gachons, C. & Breslin, P. A. Salivary amylase: digestion and metabolic syndrome. *Curr. Diab. Rep.* **16**, 102 (2016). 10.1007/s11892-016-0794-7 PMC6825871 27640169

[11] Cardello, A. Role of the human senses in food behavior. III. Taste. *Cereal Foods World* **41** 751–753 (1996).

[12] Dhillon, J., Running, C. A., Tucker, R. M. & Mattes, R. D. Effects of food form on appetite and energy balance. *Food Qual. Prefer.* **48**, 368–375 (2016).

[13] Luckett, C. R. & Seo, H. S. Consumer attitudes toward texture and other food attributes. *J. Texture Stud.* **46**, 46–57 (2015).

[14] Nederkoorn, C., Houben, K. & Havermans, R. C. Taste the texture. The relation between subjective tactile sensitivity, mouthfeel and picky eating in young adults. *Appetite* **136**, 58–61 (2019). 10.1016/j.appet.2019.01.015 30664910

[15] Pellegrino, R. & Luckett, C. R. Aversive textures and their role in food rejection. *J. Texture Stud.* **51**, 733–741 (2020). 10.1111/jtxs.12543 32533706

[16] Ditschun, T. L. et al. Overview of mouthfeel from the perspective of sensory scientists in industry. *Compr. Rev. Food Sci. Food Saf.* **24**, e70126 (2025). 10.1111/1541-4337.70126 39970006

[17] Bédard, A. et al. Can eating pleasure be a lever for healthy eating? A systematic scoping review of eating pleasure and its links with dietary behaviors and health. *PLoS ONE* **15**, e0244292 (2020). 10.1371/journal.pone.0244292 PMC7751982 33347469

[18] Moreno Rodríguez, L., Fernández Martín, A. & Díaz Armas, R. Popularizing wine tasting evaluation: an adaptation of mouthfeel terminology. *Foods* **15**, 1302 (2026). 10.3390/foods15081302 PMC13114943 42073190

[19] Imamura, E. et al. Beyond taste: multisensory aspects to food enjoyment. *Can. J. Undergrad. Res.* **10**, 40–45 (2025).

[20] Rakha, A. et al. Insights into the constellating drivers of satiety impacting dietary patterns and lifestyle. *Front. Nutr.* **9**, 1002619 (2022). 10.3389/fnut.2022.1002619 PMC9549911 36225863

[21] Bolhuis, D. P. & Forde, C. G. Application of food texture to moderate oral processing behaviors and energy intake. *Trends Food Sci. Technol.* **106**, 445–456 (2020).

[22] Chambers, L., McCrickerd, K. & Yeomans, M. R. Optimising foods for satiety. *Trends Food Sci. Technol.* **41**, 149–160 (2015).

[23] Forde, C. G., Mars, M. & De Graaf, K. Ultra-processing or oral processing? A role for energy density and eating rate in moderating energy intake from processed foods. *Curr. Dev. Nutr.* **4**, nzaa019 (2020). 10.1093/cdn/nzaa019 PMC7042610 32110771

[24] Teo, P. S., Van Dam, R. M., Whitton, C., Tan, L. W. L. & Forde, C. G. Consumption of foods with higher energy intake rates is associated with greater energy intake, adiposity, and cardiovascular risk factors in adults. *J. Nutr.* **151**, 370–378 (2021). 10.1093/jn/nxaa344 33244595

[25] Cox, D. N., Hendrie, G. A., Lease, H. J., Rebuli, M. A. & Barnes, M. How does fatty mouthfeel, saltiness or sweetness of diets contribute to dietary energy intake? *Appetite* **131**, 36–43 (2018). 10.1016/j.appet.2018.08.039 30176297

[26] Forde, C. G. & de Graaf, K. Influence of sensory properties in moderating eating behaviors and food intake. *Front. Nutr.* **9**, 841444 (2022). 10.3389/fnut.2022.841444 PMC8899294 35265658

[27] Blankenship, M. L., Grigorova, M., Katz, D. B. & Maier, J. X. Retronasal odor perception requires taste cortex, but orthonasal does not. *Curr. Biol.* **29**, 62–69 (2019). 10.1016/j.cub.2018.11.011 PMC6604050 30581018

[28] Shepherd, G. M. Smell images and the flavour system in the human brain. *Nature* **444**, 316–321 (2006). 10.1038/nature05405 17108956

[29] Squier, C. A. & Kremer, M. J. Biology of oral mucosa and esophagus. *JNCI Monogr.* **2001**, 7–15 (2001). 10.1093/oxfordjournals.jncimonographs.a003443 11694559

[30] Simons, C. T., Klein, A. H. & Carstens, E. Chemogenic subqualities of mouthfeel. *Chem. Senses* **44**, 281–288 (2019). 10.1093/chemse/bjz016 PMC6538946 31039245

[31] Moayedi, Y., Michlig, S., Park, M., Koch, A. & Lumpkin, E. A. Somatosensory innervation of healthy human oral tissues. *J. Comp. Neurol.* **529**, 3046–3061 (2021). 10.1002/cne.25148 PMC10052750 33786834

[32] Miles, L., Powell, J., Kozak, C. & Song, Y. Mechanosensitive ion channels, axonal growth, and regeneration. *Neuroscientist* **29**, 421–444 (2023). 10.1177/10738584221088575 PMC9556659 35414308

[33] Griffin, C. The fine structure of nerve endings in human buccal mucosa. *Arch. Oral. Biol.* **22**, 429–435 (1977). 10.1016/0003-9969(77)90123-6 271486

[34] Zimmerman, A., Bai, L. & Ginty, D. D. The gentle touch receptors of mammalian skin. *Science* **346**, 950–954 (2014). 10.1126/science.1254229 PMC4450345 25414303

[35] Neubarth, N. L. et al. Meissner corpuscles and their spatially intermingled afferents underlie gentle touch perception. *Science* **368**, eabb2751 (2020). 10.1126/science.abb2751 PMC7354383 32554568

[36] Moayedi, Y., Michlig, S., Park, M., Koch, A. & Lumpkin, E. A. Localization of TRP channels in healthy oral mucosa from human donors. *eNeuro* **9**, ENEURO.0328-21.2022 (2022). 10.1523/ENEURO.0328-21.2022 PMC9797210 36635242

[37] Sasase, T., Fatchiyah, F. & Ohta, T. Transient receptor potential vanilloid (TRPV) channels: basal properties and physiological potential. *Gen. Physiol. Biophys.* **41**, 165–190 (2022). 10.4149/gpb_2022016 35615999

[38] McKemy, D. D., Neuhausser, W. M. & Julius, D. Identification of a cold receptor reveals a general role for TRP channels in thermosensation. *Nature* **416**, 52–58 (2002). 10.1038/nature719 11882888

[39] Yin, Y. & Lee, S.-Y. Current view of ligand and lipid recognition by the menthol receptor TRPM8. *Trends Biochem. Sci.* **45**, 806–819 (2020). 10.1016/j.tibs.2020.05.008 PMC9284947 32532587

[40] Handler, A. & Ginty, D. D. The mechanosensory neurons of touch and their mechanisms of activation. *Nat. Rev. Neurosci.* **22**, 521–537 (2021). 10.1038/s41583-021-00489-x PMC8485761 34312536

[41] Tong, C. K., Moayedi, Y. & Lumpkin, E. A. Merkel cells and keratinocytes in oral mucosa are activated by mechanical stimulation. *Physiol. Rep.* **12**, e15826 (2024). 10.14814/phy2.15826 PMC10800296 38246872

[42] Soattin, L. et al. The biophysics of piezo1 and piezo2 mechanosensitive channels. *Biophys. Chem.* **208**, 26–33 (2016). 10.1016/j.bpc.2015.06.013 26259784

[43] Wu, Y., Zheng, J., Huang, M., Zhao, D. & Sun, B. Receptor structure-function coupling orchestrates the perception of food-derived sensory cues: a molecular bridge for food sensory perception. *Food Wellness* **2**, 100024 (2026).

[44] Halata, Z., Grim, M. & Baumann, K. I. Current understanding of Merkel cells, touch reception and the skin. *Expert Rev. Dermatol.* **5**, 109–116 (2010).

[45] Maeda, T., Ochi, K., Nakakura-Ohshima, K., Youn, S. & Wakisaka, S. The Ruffini ending as the primary mechanoreceptor in the periodontal ligament: its morphology, cytochemical features, regeneration, and development. *Crit. Rev. Oral. Biol. Med.* **10**, 307–327 (1999). 10.1177/10454411990100030401 10759411

[46] Kutter, A., Hanesch, C., Rauh, C. & Delgado, A. Impact of proprioception and tactile sensations in the mouth on the perceived thickness of semi-solid foods. *Food Qual. Prefer.* **22**, 193–197 (2011).

[47] Corey, D. P. New TRP channels in hearing and mechanosensation. *Neuron* **39**, 585–588 (2003). 10.1016/s0896-6273(03)00505-1 12925273

[48] Zhang, M. et al. TRP (transient receptor potential) ion channel family: structures, biological functions and therapeutic interventions for diseases. *Signal Transduct. Target. Ther.* **8**, 261 (2023). 10.1038/s41392-023-01464-x PMC10319900 37402746

[49] Tsiokas, L. Function and regulation of TRPP2 at the plasma membrane. *Am. J. Physiol. Ren. Physiol.* **297**, F1–F9 (2009). 10.1152/ajprenal.90277.2008 PMC2711710 19244406

[50] Chai, Z., Chen, Y. & Wang, C. β-arrestin-1: bridging GPCRs to active TRP channels. *Channels* **11**, 357–359 (2017). 10.1080/19336950.2017.1324003 PMC5626421 28459611

[51] Ranade, S. S., Syeda, R. & Patapoutian, A. Mechanically activated ion channels. *Neuron* **87**, 1162–1179 (2015). 10.1016/j.neuron.2015.08.032 PMC4582600 26402601

[52] Myers, T. A., Krimm, R. F. & Breza, J. M. Response characteristics of oral mechanosensory neurons in the mouse trigeminal ganglion. *Chem. Senses* **50**, bjaf047 (2025). 10.1093/chemse/bjaf047 PMC13204983 41131695

[53] Kim, H. K., Chung, K. -m, Xing, J., Kim, H. Y. & Youn, D. -h The trigeminal sensory system and orofacial pain. *Int. J. Mol. Sci.* **25**, 11306 (2024). 10.3390/ijms252011306 PMC11508441 39457088

[54] Takemura, M., Sugiyo, S., Moritani, M., Kobayashi, M. & Yonehara, N. Mechanisms of orofacial pain control in the central nervous system. *Arch. Histol. Cytol.* **69**, 79–100 (2006). 10.1679/aohc.69.79 16819148

[55] Henssen, D. J. H. A. et al. New insights in trigeminal anatomy: a double orofacial tract for nociceptive input. *Front. Neuroanat.* **ume 10**, 2016 (2016). 10.3389/fnana.2016.00053 PMC4861896 27242449

[56] Cerkevich, C. M., Qi, H. X. & Kaas, J. H. Thalamic input to representations of the teeth, tongue, and face in somatosensory area 3b of macaque monkeys. *J. Comp. Neurol.* **521**, 3954–3971 (2013). 10.1002/cne.23386 PMC3893768 23873330

[57] Cerkevich, C. M., Qi, H. X. & Kaas, J. H. Corticocortical projections to representations of the teeth, tongue, and face in somatosensory area 3b of macaques. *J. Comp. Neurol.* **522**, 546–572 (2014). 10.1002/cne.23426 PMC4189840 23853118

[58] de Araujo, I. E. & Simon, S. A. The gustatory cortex and multisensory integration. *Int. J. Obes.* **33**, S34–S43 (2009). 10.1038/ijo.2009.70 PMC2726647 19528978

[59] Clemens, A. M., Fernandez Delgado, Y., Mehlman, M. L., Mishra, P. & Brecht, M. Multisensory and motor representations in rat oral somatosensory cortex. *Sci. Rep.* **8**, 13556 (2018). 10.1038/s41598-018-31710-0 PMC6131144 30201995

[60] Simons, C. T., Dessirier, J.-M., Carstens, M. I., O’Mahony, M. & Carstens, E. Neurobiological and psychophysical mechanisms underlying the oral sensation produced by carbonated water. *J. Neurosci.* **19**, 8134–8144 (1999). 10.1523/JNEUROSCI.19-18-08134.1999 PMC6782458 10479713

[61] Wei, F., Wang, J., Luo, L., Tayyab Rashid, M. & Zeng, L. The perception and influencing factors of astringency, and health-promoting effects associated with phytochemicals: a comprehensive review. *Food Res. Int.* **170**, 112994 (2023). 10.1016/j.foodres.2023.112994 37316067

[62] Acevedo-Fani, A. & Singh, H. Biophysical insights into modulating lipid digestion in food emulsions. *Prog. Lipid Res.* **85**, 101129 (2022). 10.1016/j.plipres.2021.101129 34710489

[63] Voigt, N. et al. The role of lipolysis in human orosensory fat perception. *J. Lipid Res.* **55**, 870–882 (2014). 10.1194/jlr.M046029 PMC3995465 24688103

[64] Stewart, J. E. et al. Oral sensitivity to fatty acids, food consumption and BMI in human subjects. *Br. J. Nutr.* **104**, 145–152 (2010). 10.1017/S0007114510000267 20196892

[65] Pepino, M. Y., Love-Gregory, L., Klein, S. & Abumrad, N. A. The fatty acid translocase gene CD36 and lingual lipase influence oral sensitivity to fat in obese subjects. *J. Lipid Res.* **53**, 561–566 (2012). 10.1194/jlr.M021873 PMC3276480 22210925

[66] Kulkarni, B. V. & Mattes, R. D. Lingual lipase activity in the orosensory detection of fat by humans. *Am. J. Physiol. Regul. Integr. Comp. Physiol.* **306**, R879–R885 (2014). 10.1152/ajpregu.00352.2013 PMC4159735 24694384

[67] Akhtar, M., Stenzel, J., Murray, B. S. & Dickinson, E. Factors affecting the perception of creaminess of oil-in-water emulsions. *Food Hydrocoll.* **19**, 521–526 (2005).

[68] Grabenhorst, F. & Rolls, E. T. The representation of oral fat texture in the human somatosensory cortex. *Hum. Brain Mapp.* **35**, 2521–2530 (2014). 10.1002/hbm.22346 PMC6869146 24038614

[69] Rolls, E. T., Critchley, H. D., Browning, A. S., Hernadi, I. & Lenard, L. Responses to the sensory properties of fat of neurons in the primate orbitofrontal cortex. *J. Neurosci.* **19**, 1532–1540 (1999). 10.1523/JNEUROSCI.19-04-01532.1999 PMC6786033 9952429

[70] Rolls, E. T., Mills, T., Norton, A. B., Lazidis, A. & Norton, I. T. The neuronal encoding of oral fat by the coefficient of sliding friction in the cerebral cortex and amygdala. *Cereb. Cortex* **28**, 4080–4089 (2018). 10.1093/cercor/bhy213 PMC6188542 30169795

[71] Roque, J., Auvray, M. & Lafraire, J. Understanding freshness perception from the cognitive mechanisms of flavor: the case of beverages. *Front. Psychol.* **8**, 2360 (2018). 10.3389/fpsyg.2017.02360 PMC5769326 29375453

[72] Slocombe, B., Carmichael, D. & Simner, J. Cross-modal tactile–taste interactions in food evaluations. *Neuropsychologia* **88**, 58–64 (2016). 10.1016/j.neuropsychologia.2015.07.011 PMC5072487 26169315

[73] Pistolas, E. & Wagemans, J. Crossmodal correspondences and interactions between texture and taste perception. *i-Percept.* **14**, 20416695231163473 (2023). 10.1177/20416695231163473 PMC10069003 37020456

[74] Pires, M. A., Pastrana, L. M., Fuciños, P., Abreu, C. S. & Oliveira, S. M. Sensorial perception of astringency: oral mechanisms and current analysis methods. *Foods* **9**, 1124 (2020). 10.3390/foods9081124 PMC7465539 32824086

[75] Roque, J., Lafraire, J. & Auvray, M. Audiovisual crossmodal correspondence between bubbles’ size and pouring sounds’ pitch in carbonated beverages. *Foods* **9**, 966 (2020). 10.3390/foods9080966 PMC7466368 32707765

[76] Zampini, M. & Spence, C. The role of auditory cues in modulating the perceived crispness and staleness of potato chips. *J. Sens. Stud.* **19**, 347–363 (2004).

[77] Nishinari, K. et al. The role of texture in the palatability and food oral processing. *Food Hydrocoll.* **147**, 109095 (2024).

[78] Lederman, S. J., Thorne, G. & Jones, B. Perception of texture by vision and touch: multidimensionality and intersensory integration. *J. Exp. Psychol. Hum. Percept. Perform.* **12**, 169–180 (1986). 10.1037//0096-1523.12.2.169 2940321

[79] Chylinski, M., Northey, G. & Ngo, L. V. Cross-modal interactions between color and texture of food. *Psychol. Mark.* **32**, 950–966 (2015).

[80] Rohm, H., Strobl, M. & Jaros, D. Butter colour affects sensory perception of spreadability. *Z. f.ür. Lebensmitteluntersuchung und -Forsch. A* **205**, 108–110 (1997).

[81] Kangur, K., Giesel, M., Harris, J. M. & Hesse, C. Crossmodal texture perception is illumination-dependent. *Multisens. Res.* **36**, 75–91 (2022). 10.1163/22134808-bja10089 36731529

[82] Eck, J., Kaas, A. L. & Goebel, R. Crossmodal interactions of haptic and visual texture information in early sensory cortex. *Neuroimage* **75**, 123–135 (2013). 10.1016/j.neuroimage.2013.02.075 23507388

[83] Klosse, P. *The Essence of Gastronomy: Understanding the Flavor of Foods and Beverages* (CRC Press, 2013).

[84] van der Stelt, A. J., Mehring, P., Corbier, C., van Eijnatten, E. J. M. & Withers, C. A mouthfeel wheel” terminology for communicating the mouthfeel attributes of medical nutrition products (MNP). *Food Qual. Prefer.* **80**, 103822 (2020).

[85] Yu, M. et al. Flavor wheel construction and sensory profile description of human milk. *Nutrients* **14**, 5387 (2022). 10.3390/nu14245387 PMC9783944 36558546

[86] Suwonsichon, S. The importance of sensory lexicons for research and development of food products. *Foods* **8**, 27 (2019). 10.3390/foods8010027 PMC6352027 30650601

[87] Agorastos, G., van E. H., Bast, A. & Klosse, P. Review of mouthfeel classification. a new perspective on food perception. *J. Food. Sci. Nutr*. **14**, 1–10 (2020).

[88] Drake, B. Sensory textural/rheological properties—a polyglot list. *J. Texture Stud.* **20**, 1–27 (1989).

[89] Neiers, F. et al. Food formulation: rheological and tribological determinants of oral processing and flavor perception. *Food Res. Int.* **228**, 118377 (2026). 10.1016/j.foodres.2026.118377 41703839

[90] Koehler, M. et al. Biophysical investigations using atomic force microscopy can elucidate the link between mouthfeel and flavour perception. *Nat. Food* **5**, 281–287 (2024). 10.1038/s43016-024-00958-3 38605131

[91] Andrewes, P., Bullock, S., Turnbull, R. & Coolbear, T. Chemical instrumental analysis versus human evaluation to measure sensory properties of dairy products: what is fit for purpose? *Int. Dairy J.* **121**, 105098 (2021).

[92] Teo, P. S., van Dam, R. M., Whitton, C., Tan, L. W. L. & Forde, C. G. Association between self-reported eating rate, energy intake, and cardiovascular risk factors in a multi-ethnic Asian population. *Nutrients* **12**, 1080 (2020). 10.3390/nu12041080 PMC7230501 32295057

[93] Nguyen, Q. C. & Varela, P. in *Food Texturology: Measurement and Perception of Food Textural Properties* (Springer, 2023).

[94] Foster, K. D. et al. The role of oral processing in dynamic sensory perception. *J. Food Sci.* **76**, R49–R61 (2011). 10.1111/j.1750-3841.2010.02029.x 21535784

[95] Pellegrino, R. et al. The contribution of texture contrasts and combinations to food acceptance across cultures. *J. Texture Stud.* **51**, 225–231 (2020). 10.1111/jtxs.12494 31697403

[96] Jeltema, M., Beckley, J. & Vahalik, J. Food texture assessment and preference based on mouth behavior. *Food Qual. Prefer.* **52**, 160–171 (2016).

[97] Palczak, J., Giboreau, A., Rogeaux, M. & Delarue, J. How do pastry and culinary chefs design sensory complexity? *Int. J. Gastron. Food Sci.* **19**, 100182 (2020).

[98] Kumar, R. & Chambers, E. Understanding the terminology for snack foods and their texture by consumers in four languages: a qualitative study. *Foods* **8**, 484 (2019). 10.3390/foods8100484 PMC6835815 31614755

[99] Gupta, M. K. et al. Plant and dairy-based yogurts: a comparison of consumer sensory acceptability linked to textural analysis. *Foods* **11**, 463 (2022). 10.3390/foods11030463 PMC8834546 35159613

[100] Falkeisen, A. et al. Consumer perception and emotional responses to plant-based cheeses. *Food Res. Int.* **158**, 111513 (2022). 10.1016/j.foodres.2022.111513 35840221

[101] Wahlqvist, M. L. Food structure is critical for optimal health. *Food Funct.* **7**, 1245–1250 (2016). 10.1039/c5fo01285f 26667120

[102] Rolls, E. T. Taste, olfactory, and food texture processing in the brain, and the control of food intake. *Physiol. Behav.* **85**, 45–56 (2005). 10.1016/j.physbeh.2005.04.012 15924905

[103] Steptoe, A. Development of a measure of the motives underlying the selection of food: the food choice questionnaire. *Appetite* **25**, 267–284 (1995). 10.1006/appe.1995.0061 8746966

[104] de Graaf, C. et al. Food acceptability in field studies with US Army men and women: relationship with food intake and food choice after repeated exposures. *Appetite* **44**, 23–31 (2005). 10.1016/j.appet.2004.08.008 15604031

[105] Gallego, M., Barat, J. M., Grau, R. & Talens, P. Compositional, structural design and nutritional aspects of texture-modified foods for the elderly. *Trends Food Sci. Technol.* **119**, 152–163 (2022).

[106] Hazley, D., Stack, M., Walton, J., McNulty, B. A. & Kearney, J. M. Food neophobia across the life course: pooling data from five national cross-sectional surveys in Ireland. *Appetite* **171**, 105941 (2022). 10.1016/j.appet.2022.105941 35066004

[107] Chow, C. Y., Skouw, S., Bech, A. C., Olsen, A. & Bredie, W. L. A review on children’s oral texture perception and preferences in foods. *Crit. Rev. Food Sci. Nutr.* **64**, 3861–3879 (2024). 10.1080/10408398.2022.2136619 36300653

[108] Carruth, B. R. & Skinner, J. D. Revisiting the picky eater phenomenon: neophobic behaviors of young children. *J. Am. Coll. Nutr.* **19**, 771–780 (2000). 10.1080/07315724.2000.10718077 11194531

[109] Cooke, L., Wardle, J. & Gibson, E. Relationship between parental report of food neophobia and everyday food consumption in 2–6-year-old children. *Appetite* **41**, 205–206 (2003). 10.1016/s0195-6663(03)00048-5 14550320

[110] Falciglia, G. A., Couch, S. C., Gribble, L. S., Pabst, S. M. & Frank, R. Food neophobia in childhood affects dietary variety. *J. Am. Diet. Assoc.* **100**, 1474–1481 (2000). 10.1016/S0002-8223(00)00412-0 11138439

[111] Hall, K. D. et al. Ultra-processed diets cause excess calorie intake and weight gain: an inpatient randomized controlled trial of ad libitum food intake. *Cell Metab.* **30**, 67–77 (2019). 10.1016/j.cmet.2019.05.008 PMC7946062 31105044

[112] Knaapila, A. J. et al. Food neophobia associates with lower dietary quality and higher BMI in Finnish adults. *Public Health Nutr.* **18**, 2161–2171 (2015). 10.1017/S1368980014003024 PMC10271395 25529274

[113] Hotaling, D. L. Nutritional considerations for the pureed diet texture in dysphagic elderly. *Dysphagia* **7**, 81–85 (1992). 10.1007/BF02493438 1572230

[114] Wright, L., Cotter, D., Hickson, M. & Frost, G. Comparison of energy and protein intakes of older people consuming a texture modified diet with a normal hospital diet. *J. Hum. Nutr. Diet.* **18**, 213–219 (2005). 10.1111/j.1365-277X.2005.00605.x 15882384

[115] Cichero, J. A. Y. Texture-modifi ed meals for hospital patients. Modifying food texture (Eds Chen, J. & Rosenthal, A.) Woodhead Publishing, 135–162 (2015).

[116] Fjaeldstad, A. W. & Smith, B. The effects of olfactory loss and parosmia on food and cooking habits, sensory awareness, and quality of life—a possible avenue for regaining enjoyment of food. *Foods* **11**, 1686 (2022). 10.3390/foods11121686 PMC9222253 35741884

[117] van Elst, J. M. et al. Subjective mouthfeel and temperature alterations in COVID-19 patients six to ten months after diagnosis. *Chemosens. Percept.* **15**, 165–174 (2022). 10.1007/s12078-022-09304-y PMC9660145 36406042

[118] Krop, E. M. et al. Influence of oral processing on appetite and food intake–a systematic review and meta-analysis. *Appetite* **125**, 253–269 (2018). 10.1016/j.appet.2018.01.018 29408331

[119] Stribiţcaia, E., Evans, C. E., Gibbons, C., Blundell, J. & Sarkar, A. Food texture influences on satiety: systematic review and meta-analysis. *Sci. Rep.* **10**, 12929 (2020). 10.1038/s41598-020-69504-y PMC7395742 32737349

[120] Appleton, K. M. et al. Sensory and physical characteristics of foods that impact food intake without affecting acceptability: Systematic review and meta-analyses. *Obes. Rev.* **22**, e13234 (2021). 10.1111/obr.13234 PMC8365638 33754456

[121] Bolhuis, D. P. et al. Slow food: sustained impact of harder foods on the reduction in energy intake over the course of the day. *PLoS ONE* **9**, e93370 (2014). 10.1371/journal.pone.0093370 PMC3973680 24695412

[122] Juvonen, K. R. et al. Viscosity of oat bran-enriched beverages influences gastrointestinal hormonal responses in healthy humans. *J. Nutr.* **139**, 461–466 (2009). 10.3945/jn.108.099945 19176745

[123] Zhu, Y., Hsu, W. H. & Hollis, J. H. The impact of food viscosity on eating rate, subjective appetite, glycemic response and gastric emptying rate. *PLoS ONE* **8**, e67482 (2013). 10.1371/journal.pone.0067482 PMC3688614 23818981

[124] Jourdren, S. et al. Breakdown pathways during oral processing of different breads: impact of crumb and crust structures. *Food Funct.* **7**, 1446–1457 (2016). 10.1039/c5fo01286d 26857555

[125] Chen, J. Food oral processing: mechanisms and implications of food oral destruction. *Trends Food Sci. Technol.* **45**, 222–228 (2015).

[126] Garutti, M. et al. Hallmarks of appetite: a comprehensive review of hunger, appetite, satiation, and satiety. *Curr. Obes. Rep.* **14**, 12 (2025). 10.1007/s13679-024-00604-w 39849268

[127] Avena, N. M., Rada, P. & Hoebel, B. G. Sugar and fat bingeing have notable differences in addictive-like behavior. *J. Nutr.* **139**, 623–628 (2009). 10.3945/jn.108.097584 PMC2714381 19176748

[128] Leonard, W. R., Snodgrass, J. J. & Robertson, M. L. in *Fat Detection: Taste, Texture, and Post Ingestive Effects* (eds Montmayeur, J. -P. & le Coutre, J.) Ch 1 (CRC Press, 2009). 21452472

[129] Lett, A. M., Norton, J. E. & Yeomans, M. R. Emulsion oil droplet size significantly affects satiety: a pre-ingestive approach. *Appetite* **96**, 18–24 (2016). 10.1016/j.appet.2015.08.043 26344811

[130] Feinle, C., O’Donovan, D. & Horowitz, M. Carbohydrate and satiety. *Nutr. Rev.* **60**, 155–169 (2002). 10.1301/002966402320243241 12078914

[131] Drewnowski, A. Energy density, palatability, and satiety: implications for weight control. *Nutr. Rev.* **56**, 347–353 (1998). 10.1111/j.1753-4887.1998.tb01677.x 9884582

[132] Rada, P., Avena, N. M. & Hoebel, B. G. Daily bingeing on sugar repeatedly releases dopamine in the accumbens shell. *Neuroscience* **134**, 737–744 (2005). 10.1016/j.neuroscience.2005.04.043 15987666

[133] De Macedo, I. C., de Freitas, J. S. & da Silva Torres, I. L. The influence of palatable diets in reward system activation: a mini review. *Adv. Pharmacol. Pharm. Sci.* **2016**, 7238679 (2016). 10.1155/2016/7238679 PMC4818794 27087806

[134] De Graaf, C., De Jong, L. S. & Lambers, A. C. Palatability affects satiation but not satiety. *Physiol. Behav.* **66**, 681–688 (1999). 10.1016/s0031-9384(98)00335-7 10386914

[135] Gibney, M. J. Food texture trumps food processing in the regulation of energy intake. *Am. J. Clin. Nutr.* **116**, 9–10 (2022). 10.1093/ajcn/nqac104 35585666

[136] Sørensen, L. B., Møller, P., Flint, A., Martens, M. & Raben, A. Effect of sensory perception of foods on appetite and food intake: a review of studies on humans. *Int. J. Obes.* **27**, 1152–1166 (2003). 10.1038/sj.ijo.0802391 14513063

[137] Chung, S.-J. & Vickers, Z. Influence of sweetness on the sensory-specific satiety and long-term acceptability of tea. *Food Qual. Prefer.* **18**, 256–264 (2007).

[138] Rossi, M. A. & Stuber, G. D. Overlapping brain circuits for homeostatic and hedonic feeding. *Cell Metab.* **27**, 42–56 (2018). 10.1016/j.cmet.2017.09.021 PMC5762260 29107504

[139] Lutter, M. & Nestler, E. J. Homeostatic and hedonic signals interact in the regulation of food intake. *J. Nutr.* **139**, 629–632 (2009). 10.3945/jn.108.097618 PMC2714382 19176746

[140] Ventura, A. K. & Birch, L. L. Does parenting affect children’s eating and weight status? *Int. J. Behav. Nutr. Phys. Act.* **5**, 15 (2008). 10.1186/1479-5868-5-15 PMC2276506 18346282

[141] Rigamonti, A. E. et al. Anticipatory and consummatory effects of (hedonic) chocolate intake are associated with increased circulating levels of the orexigenic peptide ghrelin and endocannabinoids in obese adults. *Food Nutr. Res.* **59**, 29678 (2015). 10.3402/fnr.v59.29678 PMC4636866 26546790

[142] Johnson, P. M. & Kenny, P. J. Dopamine D2 receptors in addiction-like reward dysfunction and compulsive eating in obese rats. *Nat. Neurosci.* **13**, 635–641 (2010). 10.1038/nn.2519 PMC2947358 20348917

[143] Schulte, E. M., Avena, N. M. & Gearhardt, A. N. Which foods may be addictive? The roles of processing, fat content, and glycemic load. *PLoS ONE* **10**, e0117959 (2015). 10.1371/journal.pone.0117959 PMC4334652 25692302

[144] Johnson, F. & Wardle, J. Variety, palatability, and obesity. *Adv. Nutr.* **5**, 851–859 (2014). 10.3945/an.114.007120 PMC4224225 25398751

[145] Brunstrom, J. M. Associative learning and the control of human dietary behavior. *Appetite* **49**, 268–271 (2007). 10.1016/j.appet.2006.11.007 17197053

[146] Sclafani, A. Learned controls of ingestive behaviour. *Appetite* **29**, 153–158 (1997). 10.1006/appe.1997.0120 9344424

[147] Tang, J., Larsen, D. S., Ferguson, L. R. & James, B. J. The effect of textural complexity of solid foods on satiation. *Physiol. Behav.* **163**, 17–24 (2016). 10.1016/j.physbeh.2016.04.042 27126966

[148] Robinson, E. et al. A systematic review and meta-analysis examining the effect of eating rate on energy intake and hunger. *Am. J. Clin. Nutr.* **100**, 123–151 (2014). 10.3945/ajcn.113.081745 24847856

[149] Tanihara, S. et al. Retrospective longitudinal study on the relationship between 8-year weight change and current eating speed. *Appetite* **57**, 179–183 (2011). 10.1016/j.appet.2011.04.017 21565235

[150] Ohkuma, T. et al. Association between eating rate and obesity: a systematic review and meta-analysis. *Int. J. Obes.* **39**, 1589–1596 (2015). 10.1038/ijo.2015.96 26100137

[151] Bolhuis, D. P., Lakemond, C. M., de Wijk, R. A., Luning, P. A. & de Graaf, C. Both longer oral sensory exposure to and higher intensity of saltiness decrease ad libitum food intake in healthy normal-weight men. *J. Nutr.* **141**, 2242–2248 (2011). 10.3945/jn.111.143867 22049294

[152] Campbell, C. L., Wagoner, T. B. & Foegeding, E. A. Designing foods for satiety: the roles of food structure and oral processing in satiation and satiety. *Food Struct.* **13**, 1–12 (2017).

[153] Ferriday, D. et al. Variation in the oral processing of everyday meals is associated with fullness and meal size; a potential nudge to reduce energy intake? *Nutrients* **8**, 315 (2016). 10.3390/nu8050315 PMC4882727 27213451

[154] Dassoff, E., Shireen, A. & Wright, A. Lipid emulsion structure, digestion behavior, physiology, and health: a scoping review and future directions. *Crit. Rev. Food Sci. Nutr.* **65**, 320–352 (2025). 10.1080/10408398.2023.2273448 37947287

[155] Rondinella, D. et al. The detrimental impact of ultra-processed foods on the human gut microbiome and gut barrier. *Nutrients* **17**, 859 (2025). 10.3390/nu17050859 PMC11901572 40077728

[156] Khorisantono, P. A. et al. A neural mechanism in the human orbitofrontal cortex for preferring high-fat foods based on oral texture. *J. Neurosci.* **43**, 8000–8017 (2023). 10.1523/JNEUROSCI.1473-23.2023 PMC10669766 37845034

[157] Melnikov, S. M. et al. Sustained hunger suppression from stable liquid food foams. *Obesity* **22**, 2131–2136 (2014). 10.1002/oby.20828 24990101

[158] Drewnowski, A. & Almiron-Roig, E. in *Fat Detection: Taste, Texture, and Post Ingestive Effects* (eds Montmayeur, J. P. & le Coutre, J.) (CRC Press/Taylor & Francis, 2010). 21452472

[159] Syan, V. et al. An overview on the types, applications and health implications of fat replacers. *J. Food Sci. Technol.* **61**, 27–38 (2024). 10.1007/s13197-022-05642-7 PMC10771406 38192702

[160] Peng, X. & Yao, Y. Carbohydrates as fat replacers. *Annu. Rev. Food Sci. Technol.* **8**, 331–351 (2017). 10.1146/annurev-food-030216-030034 28245156

[161] Patel, A. R., Nicholson, R. A. & Marangoni, A. G. Applications of fat mimetics for the replacement of saturated and hydrogenated fat in food products. *Curr. Opin. Food Sci.* **33**, 61–68 (2020).

[162] Marchetti, L. & Andrés, S. C. Use of nanocellulose in meat products. *Curr. Opin. Food Sci.* **38**, 96–101 (2021).

[163] Willett, W. et al. Food in the anthropocene: the EAT–Lancet Commission on healthy diets from sustainable food systems. *Lancet* **393**, 447–492 (2019). 10.1016/S0140-6736(18)31788-4 30660336

[164] Nolden, A. A. & Forde, C. G. The nutritional quality of plant-based foods. *Sustainability* **15**, 3324 (2023).

[165] Jafari, S. et al. Plant-based diets and risk of disease mortality: a systematic review and meta-analysis of cohort studies. *Crit. Rev. Food Sci. Nutr.* **62**, 7760–7772 (2022). 10.1080/10408398.2021.1918628 33951994

[166] Appiani, M., Cattaneo, C. & Laureati, M. Sensory properties and consumer acceptance of plant-based meat, dairy, fish and eggs analogs: a systematic review. *Front. Sustain. Food Syst.* **7**, 1268068 (2023).

[167] Giacalone, D., Clausen, M. P. & Jaeger, S. R. Understanding barriers to consumption of plant-based foods and beverages: insights from sensory and consumer science. *Curr. Opin. Food Sci.* **48**, 100919 (2022).

[168] Halm, J., Sahin, A. W., Nyhan, L., Zannini, E. & Arendt, E. K. Commercial egg replacers in pound cake systems: a comprehensive analysis of market trends and application. *Foods* **13**, 292 (2024). 10.3390/foods13020292 PMC10814760 38254593

[169] Boeck, T. et al. Lentil-based yogurt alternatives fermented with multifunctional strains of lactic acid bacteria—techno-functional, microbiological, and sensory characteristics. *Foods* **11**, 2013 (2022). 10.3390/foods11142013 PMC9317967 35885256

[170] Liu, Y. et al. Recent progress of plant protein-based amyloid-like nanofibrils. *Food Hydrocoll.* **160**, 110749 (2025).

[171] Parma, V. et al. More than smell—COVID-19 is associated with severe impairment of smell, taste, and chemesthesis. *Chem. Senses* **45**, 609–622 (2020). 10.1093/chemse/bjaa041 PMC7337664 32564071

[172] Van Wymelbeke, V. et al. Optimizing sensory quality and variety: an effective strategy for increasing meal enjoyment and food intake in older nursing home residents. *Appetite* **153**, 104749 (2020). 10.1016/j.appet.2020.104749 32450092

[173] Vandenberghe-Descamps, M. et al. Impact of blade tenderization, marinade and cooking temperature on oral comfort when eating meat in an elderly population. *Meat Sci.* **145**, 86–93 (2018). 10.1016/j.meatsci.2018.06.004 29909282

[174] Botinestean, C. et al. Possibilities for developing texture-modified beef steaks suitable for older consumers using fruit-derived proteolytic enzymes. *J. Texture Stud.* **49**, 256–261 (2018). 10.1111/jtxs.12305 29068048

[175] Eom, S. H. et al. Application of freeze–thaw enzyme impregnation to produce softened root vegetable foods for elderly consumers. *J. Texture Stud.* **49**, 404–414 (2018). 10.1111/jtxs.12341 29896872

[176] Park, J. J. & Lee, W. Y. Softening of lotus root and carrot using freeze-thaw enzyme infusion for texture-modified foods. *Food Biosci.* **35**, 100557 (2020).

[177] Zúñiga, R., Kulozik, U. & Aguilera, J. Ultrasonic generation of aerated gelatin gels stabilized by whey protein β-lactoglobulin. *Food Hydrocoll.* **25**, 958–967 (2011).

[178] Kew, B., Holmes, M., Stieger, M. & Sarkar, A. Review on fat replacement using protein-based microparticulated powders or microgels: a textural perspective. *Trends Food Sci. Technol.* **106**, 457–468 (2020). 10.1016/j.tifs.2020.10.032 PMC7763486 33380775

[179] Shewan, H. M. & Stokes, J. R. Review of techniques to manufacture micro-hydrogel particles for the food industry and their applications. *J. Food. Eng.* **119**, 781–792 (2013).

[180] García, J. et al. Design of novel functional food products enriched with bioactive extracts from holothurians for meeting the nutritional needs of the elderly. *LWT* **109**, 55–62 (2019).

[181] Endo, H., Ino, S. & Fujisaki, W. The effect of a crunchy pseudo-chewing sound on perceived texture of softened foods. *Physiol. Behav.* **167**, 324–331 (2016). 10.1016/j.physbeh.2016.10.001 27720736

[182] Karanth, S., Wiesenfarth, M., Benthin, J. & Koehler, M. Fava bean protein nanofibrils modulate cell membrane interfaces for biomolecular interactions as unveiled by atomic force microscopy. *Foods* **13**, 3411 (2024). 10.3390/foods13213411 PMC11545818 39517195

[183] Şahan, M. A discipline examining the role of senses in flavor perception: neurogastronomy. *J. Tour. Res. Inst.* **3**, 159–166 (2022).

[184] Sadikzade, R. & Turhan, K. N. In *2024**Medical Technologies Congress* (*TIPTEKNO*) 1–4 (IEEE).

[185] Pribic, T. & Azpiroz, F. Biogastronomy: factors that determine the biological response to meal ingestion. *Neurogastroenterol. Motil.* **30**, e13309 (2018). 10.1111/nmo.13309 29392797

[186] Kogut-Günthel, M. M. et al. The path to the G protein-coupled receptor structural landscape: major milestones and future directions. *Br. J. Pharmacol.* **182**, 3225–3248 (2024). 10.1111/bph.17314 39209310

[187] Richter, P. et al. Biomolecular and biophysical AFM probing reveals distinct binding of bitter peptide VAPFPEVF to TAS2R16 without inducing an intracellular calcium response. *Food Chem.* **484**, 144448 (2025). 10.1016/j.foodchem.2025.144448 40288211

[188] Azeem, M. et al. Revolutionizing food product development: use of AI in product formulation, sensory prediction & sustainable scaling. *Sch. Acad. J. Biosci.* **7**, 908–916 (2025).

[189] Oz, E. & Oz, F. Artificial intelligence-enabled ingredient substitution in food systems: a review and conceptual framework for sensory, functional, nutritional, and cultural optimization. *Foods* **14**, 3919 (2025). 10.3390/foods14223919 PMC12651232 41300077

[190] Cui, Z. et al. Artificial intelligence and food flavor: how AI models are shaping the future and revolutionary technologies for flavor food development. *Compr. Rev. Food Sci. Food Saf.* **24**, e70068 (2025). 10.1111/1541-4337.70068 39783879

[191] Jahanbakhshi, A., Abbaspour-Gilandeh, Y., Heidarbeigi, K., Roushani, M. & Momeny, M. Applications of electronic nose, electronic tongue and machine learning to measure the quality of ginger powder based on aroma and taste. *J. Food Eng.* **421**, 113197 (2027).

[192] Lu, L., Hu, Z., Hu, X., Li, D. & Tian, S. Electronic tongue and electronic nose for food quality and safety. *Food Res. Int.* **162**, 112214 (2022). 10.1016/j.foodres.2022.112214 36461383

[193] Tibaduiza, D. et al. Electronic tongues and noses: a general overview. *Biosensors* **14**, 190 (2024). 10.3390/bios14040190 PMC11048215 38667183

[194] Burkard, J., Shah, A. N., Harms, E. & Denkel, C. Impact of spatial distribution on the sensory properties of multiphase 3D-printed food configurations. *Food Qual. Pref.* **108**, 104850 (2023).

[195] Zhong, L. et al. Three-dimensional food printing: its readiness for a food and nutrition insecure world. *Proc. Nutr. Soc.* **82**, 468–477 (2023). 10.1017/S0029665123003002 37288524

[196] Wahlqvist, M. L. et al. A Food in health security (FIHS) platform in the Asia-Pacific region: the way forward. *Asia Pac. J. Clin. Nutr.* **18**, 688–702 (2009). 19965367

[197] Nguyen, P. T. M., Kravchuk, O., Bhandari, B. & Prakash, S. Effect of different hydrocolloids on texture, rheology, tribology and sensory perception of texture and mouthfeel of low-fat pot-set yoghurt. *Food Hydrocoll.* **72**, 90–104 (2017).

[198] Aguilera, J. M. & Park, D. J. Texture-modified foods for the elderly: status, technology and opportunities. *Trends Food Sci. Technol.* **57**, 156–164 (2016).

